# The Role of Ubiquitination and Hepatocyte Growth Factor-Regulated Tyrosine Kinase Substrate in the Degradation of the Adrenomedullin Type I Receptor

**DOI:** 10.1038/s41598-017-12585-z

**Published:** 2017-09-28

**Authors:** Benoît T. Roux, Claudia C. Bauer, Alister J. McNeish, Stephen G. Ward, Graeme S. Cottrell

**Affiliations:** 1 Cellular and Molecular Neuroscience, Reading School of Pharmacy, University of Reading, Reading, RG6 6UB UK; 2 Department of Pharmacy and Pharmacology, University of Bath, Claverton Down, Bath, BA2 7AY UK

## Abstract

Calcitonin receptor-like receptor (CLR) and the receptor activity-modifying protein 2 (RAMP2) comprise a receptor for adrenomedullin (AM). Although it is known that AM induces internalization of CLR•RAMP2, little is known about the molecular mechanisms that regulate the trafficking of CLR•RAMP2. Using HEK and HMEC-1 cells, we observed that AM-induced activation of CLR•RAMP2 promoted ubiquitination of CLR. A mutant (CLRΔ9KR), lacking all intracellular lysine residues was functional and trafficked similar to the wild-type receptor, but was not ubiquitinated. Degradation of CLR•RAMP2 and CLRΔ9KR•RAMP2 was not dependent on the duration of AM stimulation or ubiquitination and occurred via a mechanism that was partially prevented by peptidase inhibitors. Degradation of CLR•RAMP2 was sensitive to overexpression of hepatocyte growth factor-regulated tyrosine kinase substrate (HRS), but not to HRS knockdown, whereas CLRΔ9KR•RAMP2 degradation was unaffected. Overexpression, but not knockdown of HRS, promoted hyperubiquitination of CLR under basal conditions. Thus, we propose a role for ubiquitin and HRS in the regulation of AM-induced degradation of CLR•RAMP2.

## Introduction

Adrenomedullin (AM) was originally isolated from human pheochromocytoma cells and identified through its ability to generate cAMP in platelets^1^. AM is a potent vasodilatory peptide and shares homology with calcitonin gene-related peptide (CGRP) and together with calcitonin, amylin and adrenomedullin-2 (also known as intermedin) form the calcitonin family of peptides^2^. In contrast to CGRP, AM is mainly produced by non-nervous tissues including vascular smooth muscle cells and endothelial cells. The vascular endothelium constitutes a cellular barrier that plays a crucial role in the maintenance of vessel integrity and controls exchange of small solutes and macromolecules between the intravascular and interstitial space. Increased endothelial permeability is a hallmark of virtually every acute inflammatory reaction. Inflammation is typified by extravasation of fluid and plasma molecules as well as inflammatory mediators through the activated endothelium. Knockout mouse models affecting the AM system (*Adm* (AM gene), *Calcrl* (CLR gene) and *Pam* (*peptidylglycine α-amidating monooxygenase* gene; the enzyme which catalyzes the COOH-terminal amidation of peptide hormones)) all result in an extreme generalized oedema suggesting a role for AM in the stabilization of the endothelial barrier function^3–6^. Indeed, AM has been shown to stabilize endothelial barrier function *in vitro* and be protective in rodent models of sepsis^7–10^. Intriguingly, upregulation of AM has been observed in patients with sepsis and septic shock^11,12^.

AM and CGRP share many common functions in the cardiovascular system, and indeed they also share a common receptor component, namely the G protein-coupled receptor (GPCR), calcitonin receptor-like receptor (CLR)^13^. The receptors for AM and CGRP are heterodimeric complexes, comprising CLR and a single transmembrane protein, receptor activity-modifying protein (RAMP). If CLR is expressed with RAMP1, they form a high affinity receptor for CGRP. However, if CLR is expressed with RAMP2 or RAMP3, then high affinity AM receptors (AM_1_ and AM_2_ receptors, respectively) are formed^13^. The mechanisms that regulate CLR•RAMP1 and CLR•RAMP3 internalization and trafficking are better understood than those regulating CLR•RAMP2. The duration of stimulation with CGRP, determines whether CLR•RAMP1 is degraded or recycled^14^. Degradation of CLR•RAMP1 occurs in lysosomes via an ubiquitin-independent mechanism^14^. The recycling of CLR•RAMP1 depends on the cleavage of CGRP in acidified endosomes by the endosomal peptidase, endothelin-converting enzyme 1^15^. The proteolytic destruction of CGRP causes dissociation of β-arrestins from the endosomal CLR•RAMP1 complex, leaving CLR•RAMP1 free to recycle back to the cell-surface via a Rab11-dependent mechanism^14,15^. Hepatocyte growth factor-regulated tyrosine kinase substrate (HRS, also known as HGS) is part of a multi-protein complex localized to endosomal membranes^16,17^ that regulates the trafficking, degradation and recycling of many GPCRs^18–20^ and also the ubiquitination and deubiquitination of the epidermal growth factor receptor^21^. HRS also regulates the degradation and recycling of CLR•RAMP1^19^. In contrast to RAMP1 and RAMP2, RAMP3 contains a PSD-95/Discs-large/ZO-1 (PDZ) homology domain in its C-terminus. These PDZ domains are known to alter GPCR trafficking after internalization^22,23^. RAMP3 has been shown to interact with N-ethylmaleimide-sensitive factor through its PDZ domain, which prevents CLR•RAMP3 from entering a degradative pathway and instead CLR•RAMP3 is recycled back to the cell-surface^24^.

It is known that CLR•RAMP2 internalizes by a clathrin-dependent mechanism^25^. Once internalized CLR•RAMP2 colocalizes with the endosomal marker, transferrin and later is localized to acidic vesicles, presumably lysosomes^25^. However, it is not known if the duration of stimulus affects receptor fate as it does for CLR•RAMP1^14^. The forward trafficking of CLR•RAMP2 to the cell-surface is connected to the C-terminal tail of RAMP2, as its deletion results in sequestration of CLR•RAMP2 in the endoplasmic reticulum^26^.

The ubiquitination of eukaryotic GPCRs can function to facilitate internalization^27^, target the GPCR to lysosomes^28–30^ or regulate GPCR trafficking through the endocytic network and hence their rate of their proteolytic destruction^31,32^. In this study we aimed to determine if transient stimulation with AM promotes CLR•RAMP2 recycling and the molecular mechanism regulating the post-endocytic sorting of CLR•RAMP2.

## Results

### AM Induces ubiquitination of CLR, but not RAMP2

**Figure 1:**
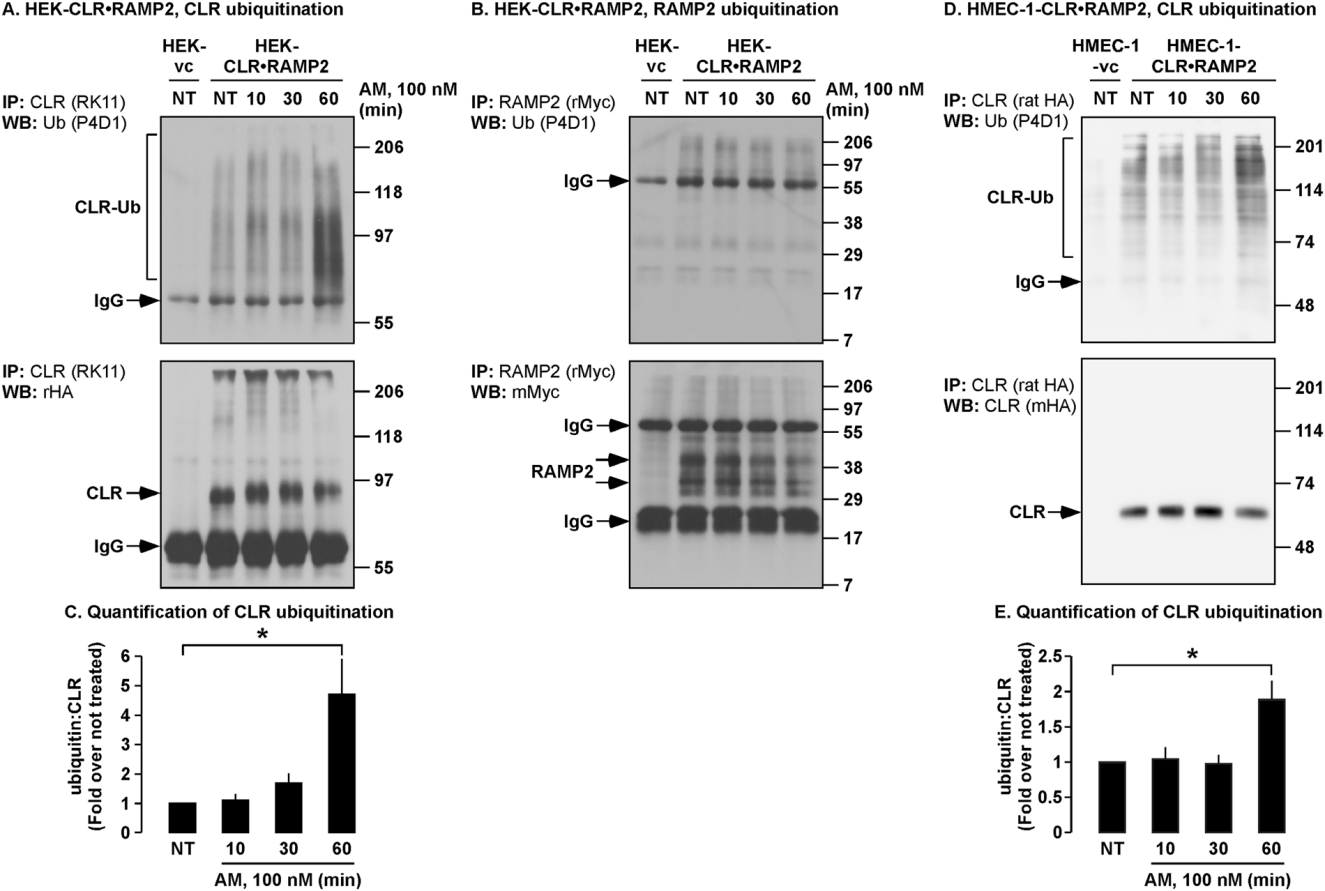
AM induces ubiquitination of CLR but not RAMP2. (**A,B,D**) HEK-CLR•RAMP2 or HMEC-1-CLR•RAMP2 cells were not treated (NT) or incubated with AM (100 nM, 0–60 min). CLR was immunoprecipitated (IP) using an antibody to CLR (RK11, HEK; rat-HA, HMEC-1) and RAMP2 was immunoprecipitated using an antibody to the extracellular epitope tag of RAMP2 (rabbit-Myc, rMyc). Immunoprecipitates were analyzed by Western blotting (WB) and probed using antibodies to ubiquitin and the extracellular epitope tags of CLR (rabbit-HA, rHA) and RAMP2 (rMyc). (**A**) In HEK-CLR•RAMP2 cells there were low levels of ubiquitinated CLR and AM induced further ubiquitination of CLR at 60 min. (**B**) AM did not induce ubiquitination of RAMP2. There were no signals for CLR and RAMP2 in vector control cells, confirming specificity of immunoprecipitating antibodies and detection. (**C**) Quantification of ubiquitinated CLR in HEK cells. (**D**) In HMEC-1-CLR•RAMP2 cells there were low levels of ubiquitinated CLR, which were increased at 60 min by challenge with AM (100 nM) (**E**) Quantification of ubiquitinated CLR in HMEC-1 cells. n = 4. ANOVA and Student-Newman-Keuls post-hoc test, *p < 0.05; HEK-vc = HEK-vector control; HMEC-1-vc = HMEC-1-vector control.

It has previously been reported that CLR•RAMP2 traffics to acidic vesicles following stimulation with AM, however the molecular mechanism regulating this trafficking is unknown^25^. Attachment of ubiquitin moieties to lysine residues is required for the targeting of some, but not all GPCRs to lysosomes^28–30,33^. To determine whether AM caused ubiquitination of CLR or RAMP2, we incubated HEK-CLR•RAMP2 cells with AM, immunoprecipitated CLR and RAMP2 and probed Western blots for CLR, RAMP2 and ubiquitin. At all time points both CLR and RAMP2 were readily detected, with no signals present in cells not expressing receptor components (Fig. 1A,B). No signals for ubiquitinated RAMP2 were detected (Fig. 1B). However, ubiquitinated CLR could readily be detected 60 min post-stimulation (Fig. 1A,C).

As AM plays an important role in the maintenance of the endothelial cell barrier^7^, we investigated whether AM also induces ubiquitination of CLR when expressed in microvascular endothelial cells. HMEC-1 cells naturally express functional CLR•RAMP2 but to facilitate biochemical studies, we virally transduced HMEC-1 cells with CLR and RAMP2 (Supplementary Fig. 1A–D). HMEC-1-CLR•RAMP2 cells were challenged with AM (0-60 min), CLR immunoprecipitated and Western blots probed for CLR and ubiquitin. CLR was readily detected, with no signals present in cells not expressing receptor components, indicating antibody specificity (Fig. 1D). AM induced ubiquitination of CLR after 60 min (Fig. 1D,E). Thus, sustained activation of CLR•RAMP2 with AM causes ubiquitination of CLR in HEK and HMEC-1 cells.

### Mutation of the intracellular lysines of CLR does not affect CLR•RAMP2 expression and internalization

As ubiquitin molecules are mainly attached to lysine residues of target proteins^34,35^, we mutated all predicted intracellular lysine residues of CLR to arginine and examined the expression, trafficking, ubiquitination and mitogenic signaling of CLRΔ9KR•RAMP2. First, we determined if mutation of the lysine residues affected expression of the receptor. We expressed CLRΔ9KR•RAMP2 in HEK cells and compared the expression with the wild-type receptor (CLR•RAMP2). An antibody to the extracellular epitope (HA) of CLR recognized single proteins of approximately the same size (~85 kDa) in both HEK-CLR•RAMP2 and HEK- CLRΔ9KR•RAMP2 cells (Supplementary Fig. 2A,B). The predicted molecular mass of CLR is 54 kDa, although there are six sites for potential N-linked glycosylation, which probably accounts for the larger apparent mass. An antibody to the extracellular epitope (Myc) of RAMP2 recognized two proteins in both HEK-CLR•RAMP2 and HEK-CLRΔ9KR•RAMP2 cells with approximate molecular masses of 32 kDa and 37 kDa (Supplementary Fig. 2A,B). The predicted molecular mass of RAMP2 is 19 kDa, thus we predict that the observed immunoreactive proteins represent unglycosylated and glycosylated dimers of RAMP2, respectively. No signals were detected in HEK-vc cells, except for β-actin, which acted as a loading control, indicating specificity of detection (Supplementary Fig. 2A,B). A requirement for a functional receptor is expression at the cell-surface. To determine if mutation of lysine residues affected cell-surface expression we simultaneously localized CLR or CLRΔ9KR and RAMP2 using antibodies to the extracellular epitope tags of the receptor components. In unstimulated cells, CLR, CLRΔ9KR and RAMP2 were present at the cell-surface. AM similarly induced trafficking of CLR with RAMP2 and CLRΔ9KR with RAMP2 to the same intracellular vesicles (Supplementary Fig. 2C,D). Together these data suggest that mutation of the intracellular facing lysine residues has no effect on the expression and internalization of CLR•RAMP2.

### Mutation of the intracellular lysines of CLR prevents ubiquitination of CLR

**Figure 2:**
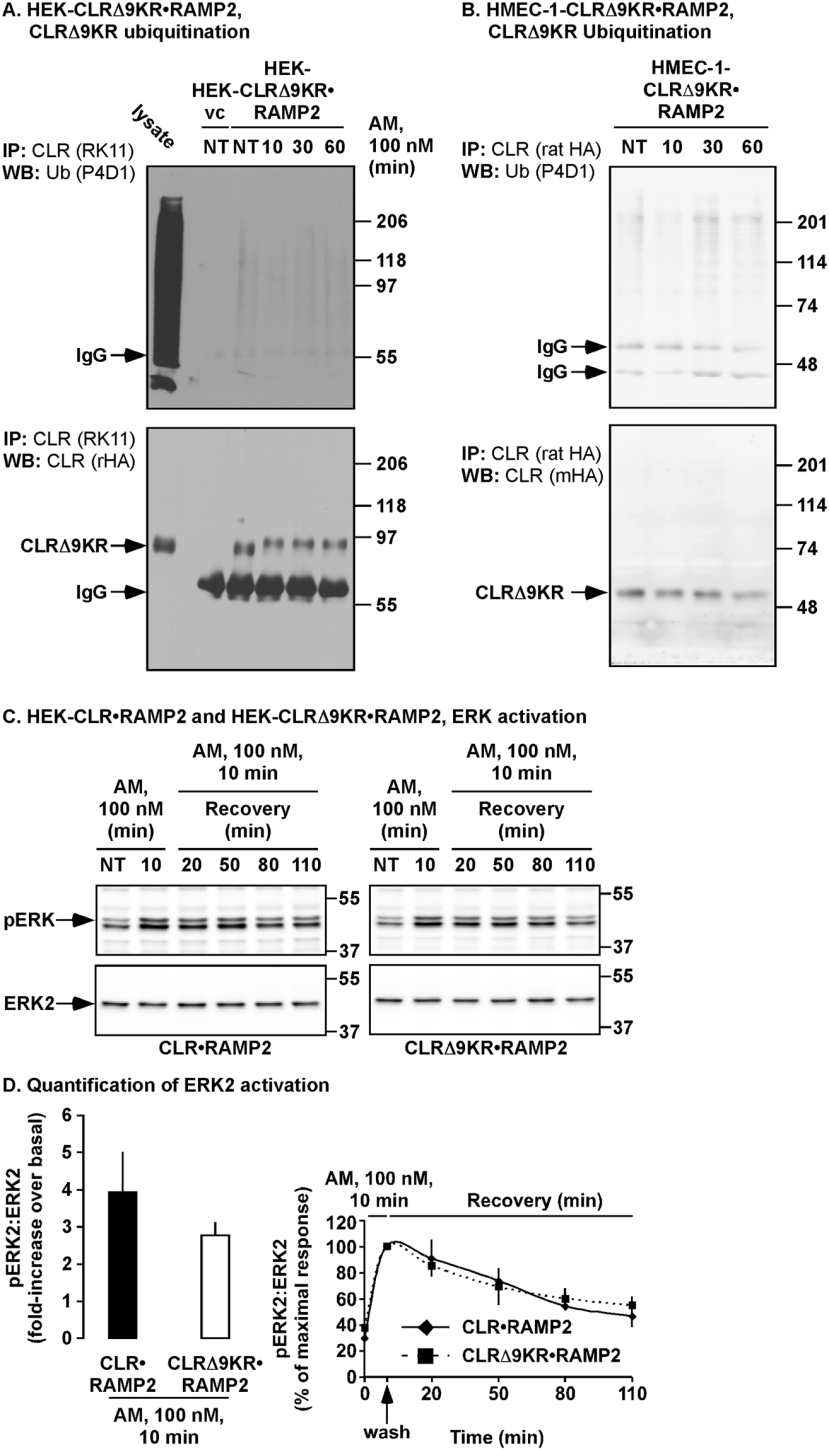
AM does not induce ubiquitination of functionally active CLRΔ9KR (**A**,**B**) HEK-CLRΔ9KR•RAMP2 or HMEC-1-CLRΔ9KR•RAMP2 cells were untreated or incubated with AM (100 nM, 0-60 min). CLRΔ9KR was immunoprecipitated (IP) using an antibody to CLR (HEK, RK11; HMEC-1, ratHA). Immunoprecipitates were analyzed by Western blotting (WB) and probed using antibodies to ubiquitin and CLR (HEK, rabbit-HA, rHA; HMEC-1, mouse-HA, mHA). AM did not induce ubiquitination of CLRΔ9KR, whereas CLRΔ9KR was readily detected by the HA antibody. There were no signals in vector control cells, confirming specificity of antibodies. n = 3–4; HEK-vc = HEK-vector control. (**C**) Serum-starved HEK-CLR•RAMP2 and HEK-CLRΔ9KR•RAMP2 cells were not treated (NT) or incubated with AM (100 nM, 0-10 min), washed and incubated in AM-free medium (0-120 min). Lysates were then analyzed for levels of phosphorylated (p) ERK2 (pERK2) and ERK2 by Western blotting. In untreated cells, levels of pERK2 were similarly low in both HEK-CLR•RAMP2 and HEK-CLRΔ9KR•RAMP2 cells. AM-induced a prompt increase in levels of pERK2 and returned towards basal levels after removal of agonist. The magnitude and duration of ERK2 activation is unaffected by the ubiquitination of CLR. (**D**) Quantification of levels of pERK1/2 in HEK-CLR•RAMP2 and HEK-CLRΔ9KR•RAMP2 cells. Results are expressed as % of maximal response (10 min, 100%). n = 3. Full length blots of panel B are shown in Supplementary Fig. 3.

Next we determined if mutation of the intracellular lysines residues affects ubiquitination of CLR. HEK- CLRΔ9KR•RAMP2 cells were stimulated with AM (0–60 min), CLR immunoprecipitated and Western blots probed for ubiquitin and CLRΔ9KR. In contrast, to CLR (Fig. 1A), no ubiquitination of CLRΔ9KR could be detected (Fig. 2A). We performed a similar experiment in HMEC-1 cells expressing CLRΔ9KR and similarly did not detect ubiquitination of CLRΔ9KR (Fig. 2B). Thus, the intracellular lysine residues are required for AM-induced ubiquitination of CLR.

### Ubiquitination has no effect on AM-induced ERK Activation

Ubiquitination of receptors and associated proteins has been shown to influence the signaling properties of activated receptors^36–38^, therefore we examined if ubiquitination of CLR regulates AM-induced ERK1/2 activation. Serum-starved HEK-CLR•RAMP2 and HEK-CLRΔ9KR•RAMP2 cells were incubated with AM (10 min), incubated in AM-free medium (0–110 min) and phosphorylated (p) and total ERK2 analyzed by Western blotting. In untreated cells, levels of pERK2 were low (Fig. 2C,D). In HEK-CLR•RAMP2 and HEK-CLRΔ9KR•RAMP2 cells, AM induced phosphorylation of ERK2 that peaked at 10 minutes and before returning back to basal. Lack of CLR ubiquitination had no effect on the magnitude of the initial and sustained activation of ERK2 (Fig. 2C,D; Supplementary Fig. 3). Thus, we conclude that ubiquitination of CLR has no role in the generation of AM-induced ERK signaling.

### Ubiquitination of CLR does not affect trafficking of CLR•RAMP2

**Figure 3:**
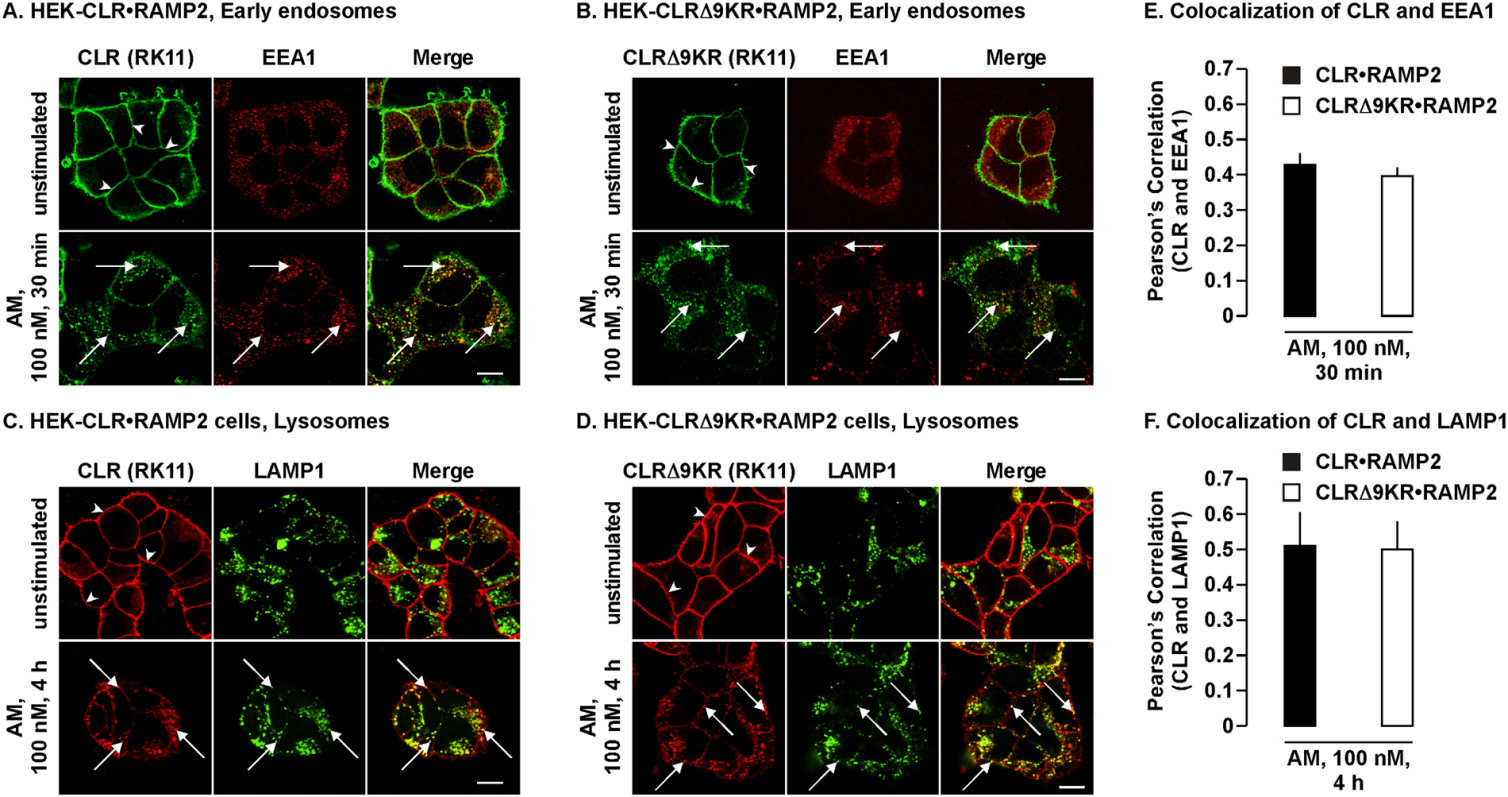
CLR and CLRΔ9KR traffic through the endocytic network to early endosomes and lysosomes. (**A,B**) HEK-CLR•RAMP2 and HEK-CLRΔ9KR•RAMP2 cells were left unstimulated or challenged with AM (100 nM, 30 min), fixed, permeabilized and CLR and a marker for early endosomes (EEA1) localized by immunofluorescence and confocal microscopy. In unstimulated cells, CLR and CLRΔ9KR were present at the cell-surface (arrowheads) and EEA1 was detected in intracellular vesicles. AM induced trafficking of CLR and CLRΔ9KR to colocalize with EEA1 in early endosomes (arrows). (**C,D**) HEK-CLR•RAMP2 and HEK-CLRΔ9KR•RAMP2 cells were incubated with lysosomal protease inhibitors, challenged with vehicle (control) or AM (100 nM, 4 h), fixed, permeabilized and CLR and a marker for lysosomes (LAMP1) localized by immunofluorescence and confocal microscopy. In unstimulated cells, CLR and CLRΔ9KR were present at the cell-surface (arrowheads) and LAMP1 was detected in intracellular vesicles. AM induced trafficking of CLR and CLRΔ9KR to colocalize with LAMP1 in lysosomes (arrows). (**E,F**) Quantification of colocalization of CLR with EEA1 and LAMP1, respectively. n = 3, Scale bar, 10 µm.

Ubiquitination of certain GPCRs is required for lysosomal targeting and degradation^28–30^, therefore determined ubiquitination of CLR affects trafficking. First, we determined if CLRΔ9KR traffics to early endosomes, similar to that reported for the wild-type receptor^25^. HEK-CLR•RAMP2 and HEK-CLRΔ9KR•RAMP2 cells were incubated with AM (30 min) and CLR and a marker for early endosomes, early endosomal antigen-1 (EEA1) localized by immunofluorescence and confocal microscopy. In unstimulated cells, both CLR and CLRΔ9KR were present at the cell-surface and EEA1 was present in intracellular vesicles (Fig. 3A,B). AM similarly induced trafficking of both CLR and CLRΔ9KR from the cell-surface to colocalize with EEA1, indicating trafficking to early endosomes (Fig. 3A,B,E). We next determined if ubiquitination of CLR is required for lysosomal targeting. HEK-CLR•RAMP2 and HEK-CLRΔ9KR•RAMP2 cells were incubated with lysosomal peptidase inhibitors, incubated with AM for 4 h and CLR and LAMP1 (a marker for lysosomes) were localized. In unstimulated cells, CLR and CLRΔ9KR were present at the cell-surface and LAMP1 was detected in intracellular vesicles (Fig. 3C,D). Incubation with AM caused internalization of CLR and CLRΔ9KR to colocalize with LAMP1, indicating trafficking to lysosomes (Fig. 3C,D,F). Thus, ubiquitination of CLR is not required for the lysosomal targeting of CLR.

### The duration of the AM challenge does not affect the trafficking or fate of activated CLR or CLRΔ9KR

**Figure 4:**
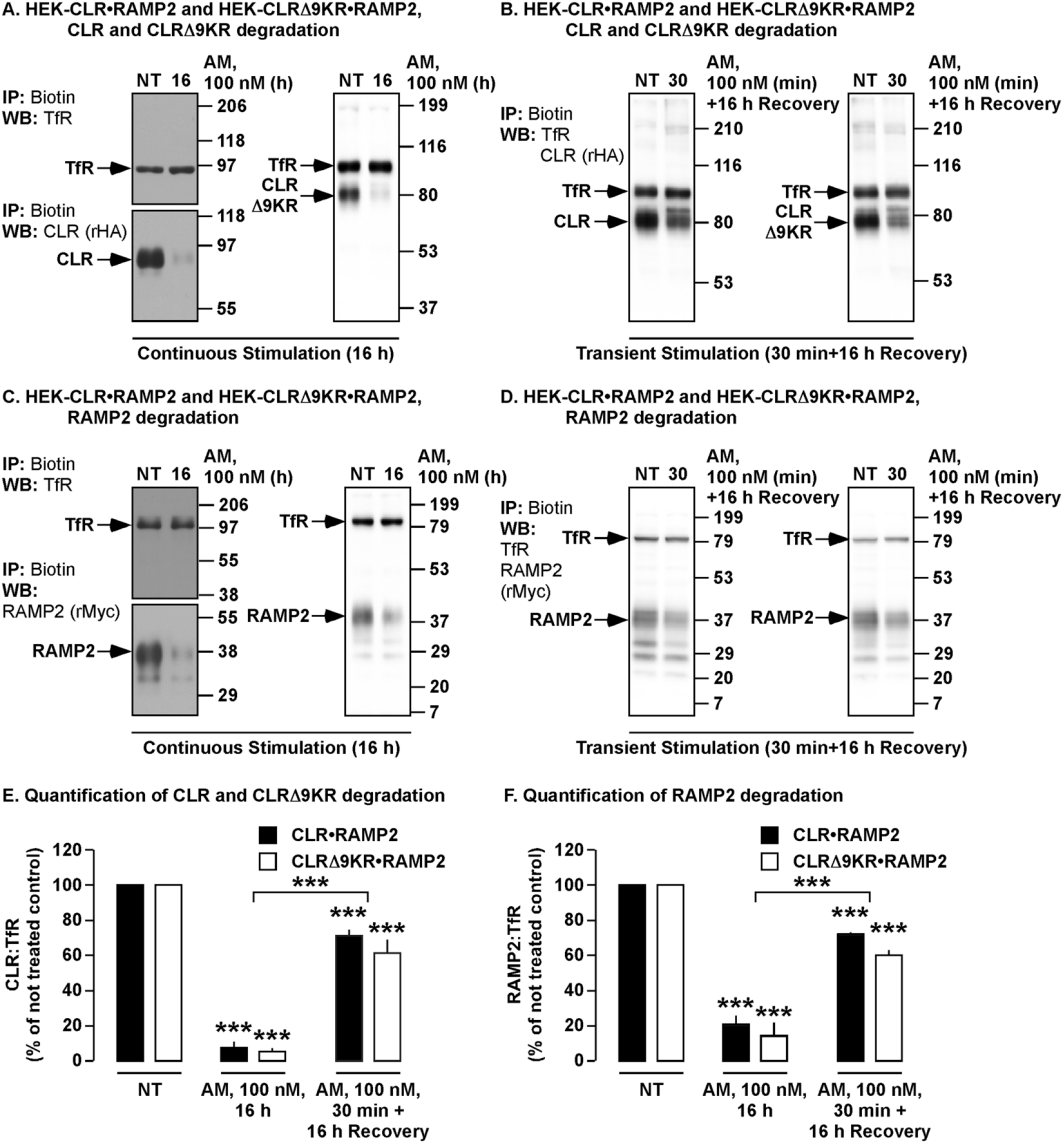
AM induces degradation of CLR, CLRΔ9KR and RAMP2 following continuous and transient challenges. Cell-surface biotinylated HEK-CLR•RAMP2 and HEK-CLRΔ9KR•RAMP2 cells were not treated (NT) or challenged with AM (100 nM, as indicated), biotinylated proteins immunoprecipitated (IP) and Western blots (WB) probed for CLR (rabbit-HA, rHA), CLRΔ9KR (rHA), RAMP2 (rMyc) and transferrin receptor (TfR, loading control). (**A,B**) In untreated HEK-CLR•RAMP2 and HEK-CLRΔ9KR•RAMP2 cells, CLR, CLRΔ9KR, RAMP2 and TfR were readily detected. AM (100 nM, 16 h) induced degradation of CLR, CLRΔ9KR and RAMP2 to similar levels. (**C,D**) In untreated HEK-CLR•RAMP2 and HEK-CLRΔ9KR•RAMP2 cells, CLR, CLRΔ9KR, RAMP2 and TfR were readily detected. AM (100 nM, 30 min, followed by 16 h incubation on AM-free medium) induced degradation of CLR, CLRΔ9KR and RAMP2 to similar levels. (**E,F)** Quantification of the degradation of CLR and CLRΔ9KR and RAMP2. n = 3–4. Data were compared by Student’s *t* test and differences among multiple groups were examined using ANOVA and Student-Newman-Keuls post-hoc test, ***p < 0.001. Full length blots of panels A and C are shown in Supplementary Fig. 4.

It has previously been shown that the trafficking and fate of the CGRP receptor (CLR•RAMP1) is dependent on the duration of stimulation^14^. To determine whether the duration of stimulation with AM similarly affects the trafficking of CLR•RAMP2, we examined levels of CLR, CLRΔ9KR and RAMP2 following either continuous or transient stimulation with AM. To examine degradation of cell-surface CLR and RAMP2, we employed a cell-surface biotinylation assay. HEK-CLR•RAMP2 and HEK-CLRΔ9KR•RAMP2 cells were challenged with AM (30 min), washed and incubated in AM-free medium for 16 h or alternatively, cells were incubated continuously with AM (16 h), biotinylated proteins immunoprecipitated and levels of CLR, CLRΔ9KR, RAMP2 and transferrin receptor (loading control) determined by Western blotting. In untreated HEK-CLR•RAMP2 and HEK-CLRΔ9KR•RAMP2 cells, CLR, CLRΔ9KR and RAMP2 were readily detected (Fig. 4A–D, Supplementary Fig. 4A,B). In contrast, to CLR•RAMP1, which only recycles following transient stimulation^14^, both transient and continuous stimulation of CLR•RAMP2 with AM caused significant degradation of CLR, CLRΔ9KR and RAMP2 (Fig. 4A–D, Supplementary Fig. 4A,B). As significantly less CLR, CLRΔ9KR and RAMP2 were degraded following a transient stimulation compared to a sustained stimulation (Fig. 4E,F), we investigated whether this was due to receptor recycling or reduced loss of cell-surface receptors. To determine the loss of cell-surface CLR•RAMP2 during the 30 minute time period we used flow cytometry. HEK-CLR•RAMP2 and HEK-CLRΔ9KR•RAMP2 cells were labeled with antibodies to the extracellular epitope of RAMP2, washed, challenged with AM (30 min) and levels of receptor remaining on the cell-surface determined. In untreated cells, levels of CLR•RAMP2 and CLRΔ9KR•RAMP2 were similar (Supplementary Fig. 5A, left panel). AM (30 min) induced a significant reduction in cell-surface expression of both CLR•RAMP2 and CLRΔ9KR•RAMP2 (Supplementary Fig. 5A, right panel; 5B, C) which is remarkably consistent with the levels of degradation determined by cell-surface biotinylation assay (Fig. 4E,F). We incubated cells for an additional period (1 h) after removal of AM and observed a further small increase in the internalization of CLR•RAMP2 and CLRΔ9KR•RAMP2 (Supplementary Fig. 5A, right panel). In a complimentary experiment, we localized CLR, CLRΔ9KR and LAMP1 by immunofluorescence and confocal microscopy. HEK-CLR•RAMP2 and HEK-CLRΔ9KR•RAMP2 cells were transiently stimulated with AM (30 min), AM removed and proteins localized after 4 h. Transient stimulation promoted the trafficking of both CLR and CLRΔ9KR to LAMP1-positive vesicles (Supplementary Fig. 6A,B). Thus, we conclude that once activated, CLR•RAMP2 is removed from the cell-surface, does not recycle to the cell-surface, traffics to LAMP1-positive vesicles and that CLR•RAMP2 and CLRΔ9KR•RAMP2 are lost from the cell-surface to similar degrees following a 30 min exposure to AM.

### Lysosomal peptidases participate in the degradation of CLR•RAMP2 and CLRΔ9KR•RAMP2

**Figure 5:**
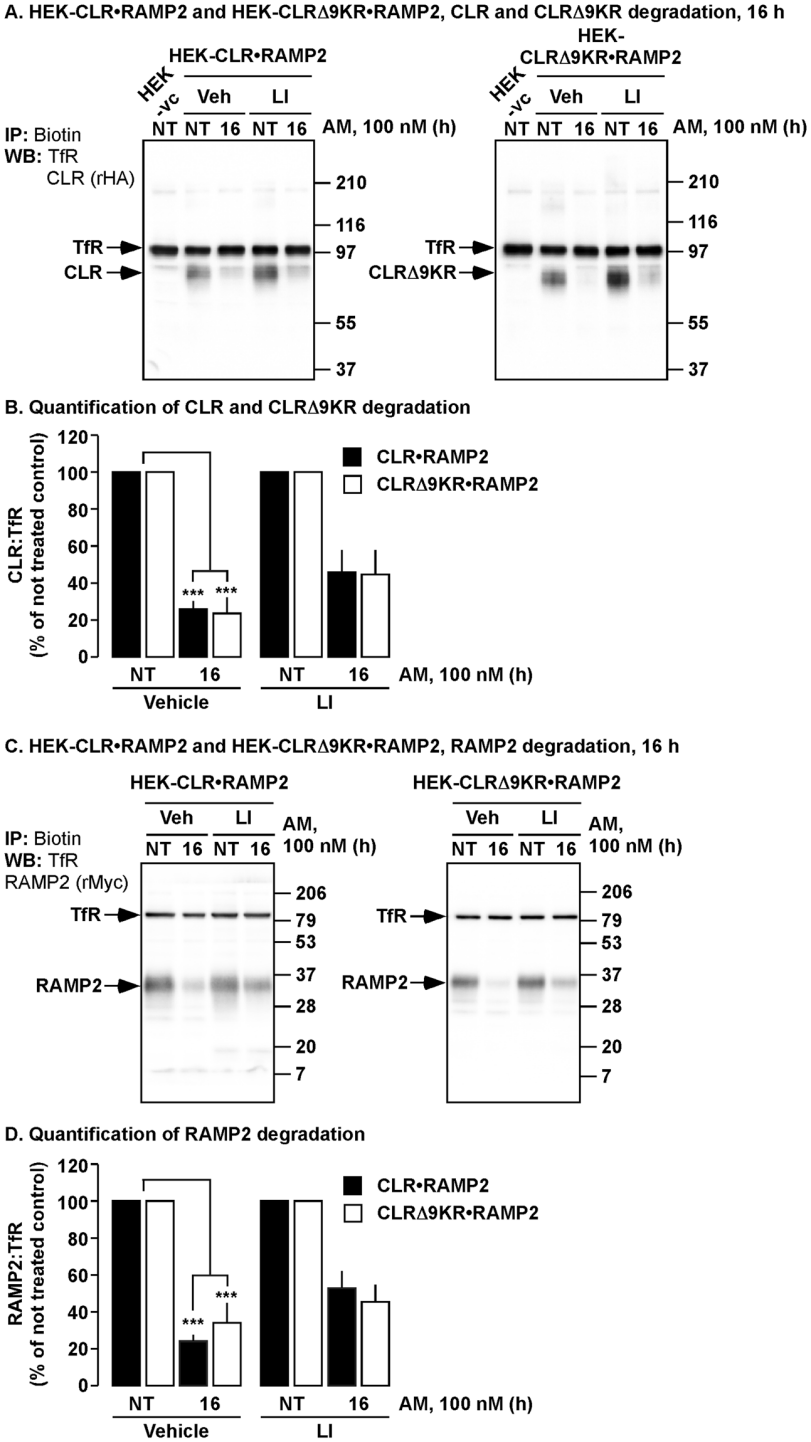
Inhibition of lysosomal peptidases partially prevents degradation of CLR•RAMP2 and CLRΔ9KR•RAMP2. (**A, C**) Cell-surface biotinylated HEK-CLR•RAMP2 and HEK-CLRΔ9KR•RAMP2 cells were incubated with vehicle (control), lysosomal peptidase inhibitors (LI), not treated (NT) or challenged with AM (100 nM, 16 h), biotinylated proteins immunoprecipitated (IP) and Western blots (WB) probed for CLR (rabbit-HA, rHA), CLRΔ9KR (rHA), RAMP2 (rabbit-Myc, rMyc) and transferrin receptor (TfR, loading control). In untreated HEK-CLR•RAMP2 and HEK-CLRΔ9KR•RAMP2 cells, CLR, CLRΔ9KR, RAMP2 and TfR were readily detected. AM (100 nM, 16 h) induced degradation of CLR, CLRΔ9KR and RAMP2 to similar levels. (**B**) Quantification of the degradation of CLR and CLRΔ9KR, respectively. (**D**) Quantification of the degradation of RAMP2. n = 4, Data were compared by Student’s *t* test, *p < 0.05, HEK-vc = HEK-vector control.

Lysosomal and proteasomal peptidases have both been implicated in the degradation of GPCRs^39,40^. Therefore, we investigated the peptidases responsible for the degradation of CLR, CLRΔ9KR and RAMP2. HEK-CLR•RAMP2 and HEK-CLRΔ9KR•RAMP2 cells were preincubated with lysosomal peptidase inhibitors or vehicle (control) and stimulated with AM. We then determined levels of CLR, CLRΔ9KR, RAMP2 and TfR by Western blotting. In vehicle-treated cells CLR, CLRΔ9KR and RAMP2 were degraded to similar levels (Fig. 5A–D). Incubation with lysosomal inhibitors partially prevented degradation of CLR, CLRΔ9KR and RAMP2 by similar amounts (Fig. 5A–D). Thus, lysosomal peptidases partially regulate the degradative process, irrespective of whether CLR is ubiquitinated or not.

To further determine if lysosomal peptidases are involved in the degradation of CLR and RAMP2, we determined the effect of the serine protease inhibitor, leupeptin an inhibitor that has been used to prevent the degradation of other GPCRs^41^. HEK-CLR•RAMP2 cells were treated as above and degradation of CLR, RAMP2 and TfR determined by Western blotting. Incubation of cells with leupeptin did not prevent the degradation of CLR and RAMP2 (Supplementary Fig. 7A–C). Although the degradation of GPCRs by the proteasome is unusual^39,40^, we investigated the role of the proteasome in the degradation of CLR•RAMP2. We quantified degradation of CLR, CLRΔ9KR and RAMP2 in the presence of the highly specific proteasome inhibitor, epoxomicin. In vehicle- and epoxomicin-treated cells, CLR, CLRΔ9KR and RAMP2 were degraded to similar levels (Supplementary Fig. 8A–D). Thus, the proteasome plays no role in the degradation of CLR, CLRΔ9KR and RAMP2.

### Kinetics of degradation of CLR•RAMP2 are not regulated by AM-induced ubiquitination

**Figure 6:**
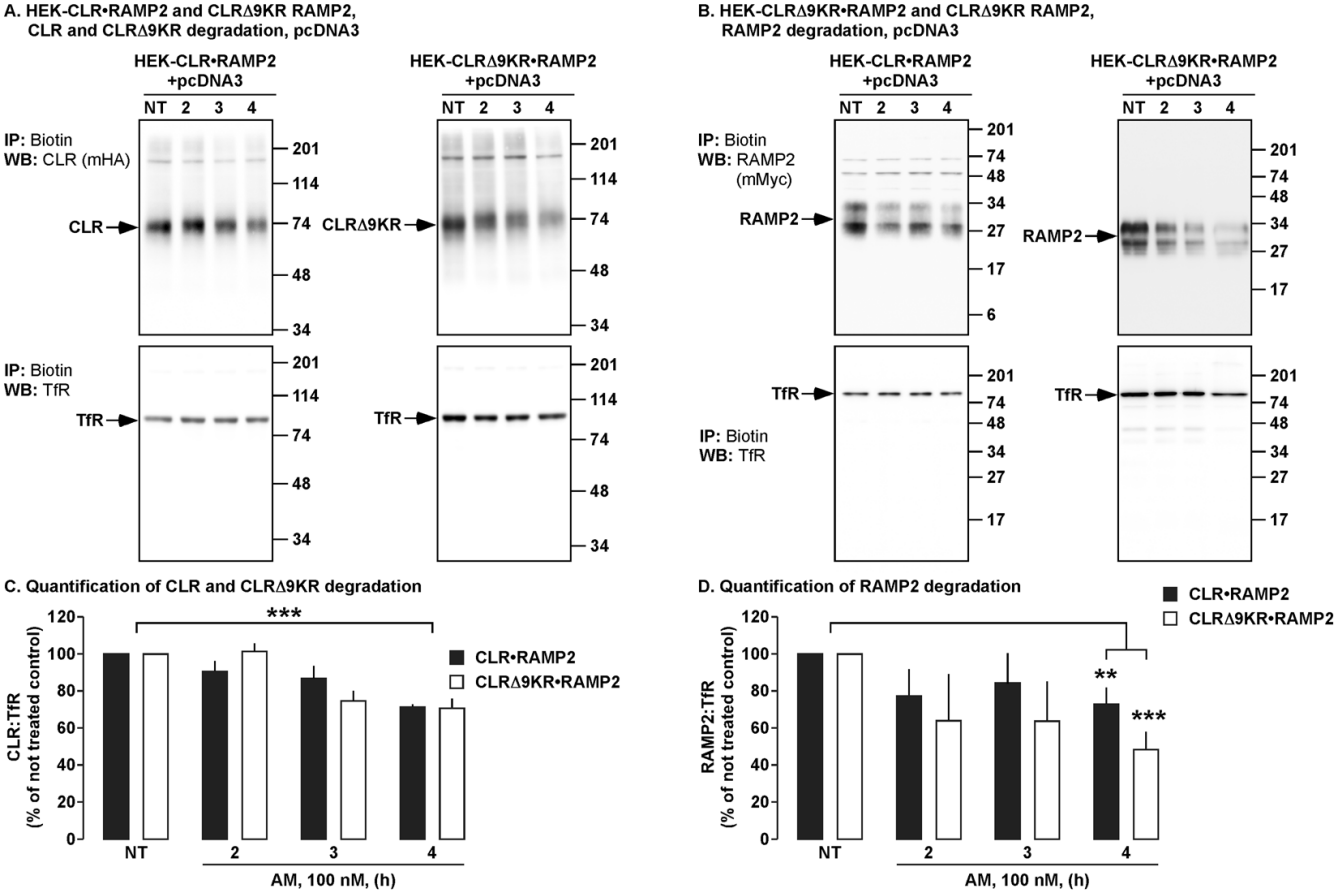
Early kinetics of CLR, CLRΔ9KR and RAMP2 degradation. (**A,B**) Cell-surface biotinylated HEK-CLR•RAMP2 and HEK-CLRΔ9KR•RAMP2 cells were not treated (NT) or challenged with AM (100 nM, 2-4 h), biotinylated proteins immunoprecipitated (IP) and Western blots (WB) probed for CLR (mouse-HA, mHA), CLRΔ9KR (mHA), RAMP2 (mouse-Myc, mMyc) and transferrin receptor (TfR, loading control). In untreated HEK-CLR•RAMP2 and HEK-CLRΔ9KR•RAMP2 cells, CLR, CLRΔ9KR, RAMP2 and TfR were readily detected. AM (100 nM, 4 h) induced degradation of CLR, CLRΔ9KR and RAMP2 to similar levels. (**C,D**) Quantification of the degradation of CLR, CLRΔ9KR and RAMP2. n ≥ 4. Data were examined using ANOVA and Student-Newman-Keuls post-hoc test, *p < 0.05, **p < 0.01, ***p < 0.001.

Ubiquitination of GPCRs has also been shown to regulate the degradation rate by altering the trafficking through the endocytic system (25,26). Therefore we determined if ubiquitination altered the rate of degradation of CLR, CLRΔ9KR and RAMP2 by examining degradation following stimulation with AM at a shorter time points. HEK-CLR•RAMP2 and HEK-CLRΔ9KR•RAMP2 cells were stimulated with AM (0–4 h) and we then determined levels of CLR, CLRΔ9KR, RAMP2 and TfR. In HEK-CLR•RAMP2 and HEK-CLRΔ9KR•RAMP2 cells, CLR, CLRΔ9KR and RAMP2 were degraded to similar levels (Fig. 6A–D). We also performed the same experiments in HMEC-1 cells and observed similar results (Supplementary Fig. 9A–C). Thus, AM-induced ubiquitination of CLR does not affect the kinetics of degradation.

### HRS regulates the degradation of ubiquitinated CLR•RAMP2, but not CLRΔ9KR•RAMP2

**Figure 7:**
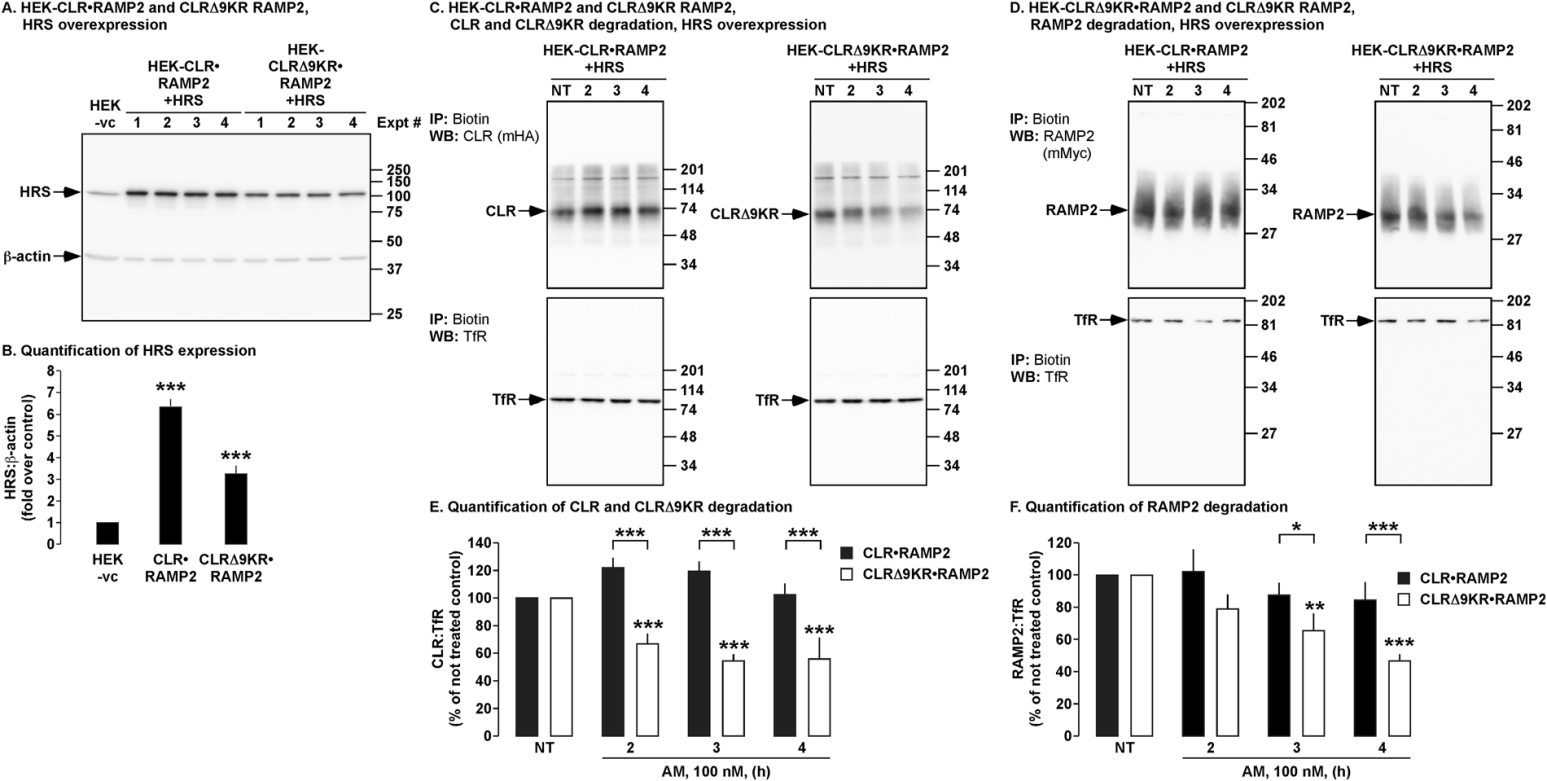
Effect of HRS on the degradation of CLR, CLRΔ9KR and RAMP2. (**A,B**) Cell lysates from experimental cells were analyzed by Western blotting and levels of HRS and β-actin quantified. (**C,D**) Cell-surface biotinylated HEK-CLR•RAMP2 and HEK-CLRΔ9KR•RAMP2 cells expressing HRS were not treated (NT) or challenged with AM (100 nM, 2-4 h), biotinylated proteins immunoprecipitated (IP) and Western blots (WB) probed for CLR (mouse-HA, mHA), CLRΔ9KR (mHA), RAMP2 (mouse-Myc, mMyc) and transferrin receptor (TfR, loading control). In untreated HEK-CLR•RAMP2 and HEK-CLRΔ9KR•RAMP2 cells, CLR, CLRΔ9KR, RAMP2 and TfR were readily detected. AM (100 nM, 4 h) induced degradation of CLR, CLRΔ9KR and RAMP2 to different levels. (**E,F**) Quantification of degradation of CLR, CLRΔ9KR and RAMP2. n = 4, Data were examined using ANOVA and Student-Newman-Keuls post-hoc test, *p < 0.05, **p < 0.01, ***p < 0.001.

HRS is an endosomal sorting protein that has previously been shown to play a role in the trafficking and degradation of CLR•RAMP1^19^. Therefore we investigated if HRS also regulates the degradation of CLR•RAMP2. We quantified the overexpression of HRS in HEK cells expressing HRS and either CLR•RAMP2 or CLRΔ9KR•RAMP2 (Fig. 7A,B). The same cells were incubated with AM (0–4 h) and levels of CLR, CLRΔ9KR and RAMP2 determined. Expression of HRS prevented the degradation of both CLR and RAMP2 in cells expressing CLR•RAMP2 (Fig. 7C–F). In contrast, expression of HRS did not affect the degradation of CLRΔ9KR and RAMP2 in CLRΔ9KR•RAMP2 cells (Fig. 7C–F). Thus, HRS is not required for the degradation of CLRΔ9KR.

### Ubiquitin-interaction motif of HRS does not regulate degradation of ubiquitinated CLR

HRS concentrates ubiquitinated receptors by direct interaction with its ubiquitin-interaction motif (UIM)^42^. Therefore we investigated if the UIM of HRS was essential for its regulation of CLR degradation. Overexpression of HRSΔUIM in HEK cells expressing HRS and either CLR•RAMP2 or CLRΔ9KR•RAMP2 was quantified by Western blotting (Supplementary Fig. 8A,B). The same cells were incubated with AM (0–4 h) and levels of CLR, CLRΔ9KR and RAMP2 quantified (Supplementary Fig. 10C–F). In HEK cells expressing HRSΔUIM and either CLR•RAMP2 or CLRΔ9KR•RAMP2 cells, we observed similar degradation profiles for CLR, CLRΔ9KR and RAMP2 as when they are co-expressed with HRS (Supplementary Fig. 10C–F). Thus, the UIM of HRS is not involved in the HRS-dependent regulation of CLR degradation.

### Knockdown of HRS does not affect AM-induced degradation of CLR•RAMP2

**Figure 8:**
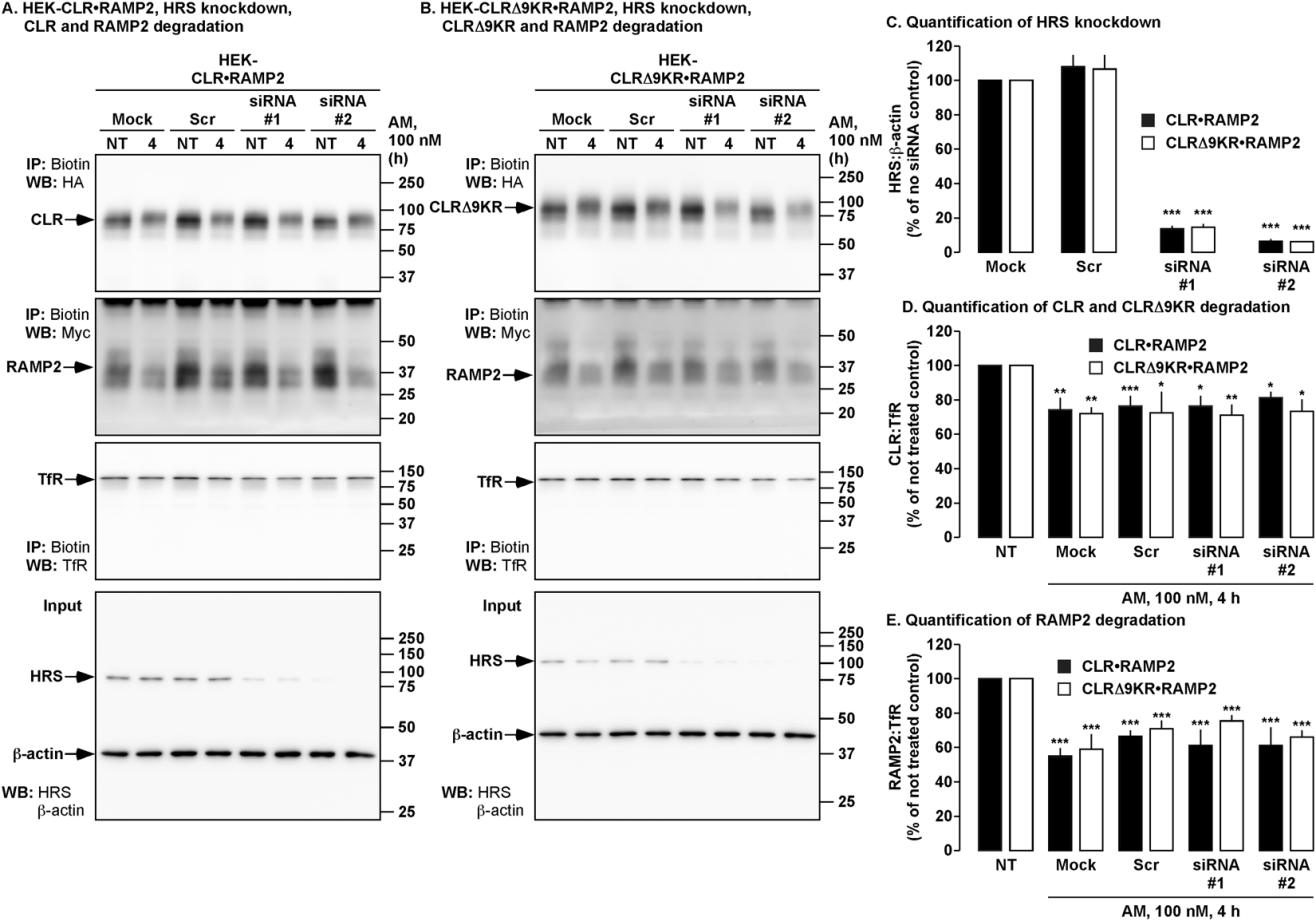
Effect of HRS knockdown on degradation of CLR, CLRΔ9KR and RAMP2. (**A,B)** Cell-surface biotinylated HEK cells expressing CLR•RAMP2 or CLRΔ9KR•RAMP2 and either mock transfected (Mock, control) or transfected with a non-targeting pool of siRNA (Scr) or siRNA to HRS (siRNA #1 and #2) were not treated (NT) or challenged with AM (100 nM, 4 h), biotinylated proteins immunoprecipitated (IP) and Western blots (WB) probed for CLR (mouse-HA, mHA), CLRΔ9KR (mHA), RAMP2 (mouse-Myc, mMyc) and transferrin receptor (TfR, loading control). In untreated HEK-CLR•RAMP2 and HEK-CLRΔ9KR•RAMP2 cells, CLR, CLRΔ9KR, RAMP2 and TfR were readily detected in all treatment types. AM (100 nM, 4 h) induced significant degradation of CLR, CLRΔ9KR and RAMP2 to similar levels in each cell type. **(C**) Quantification of the knockdown of HRS by siRNA. (**D,E**) Quantification of the degradation of CLR, CLRΔ9KR and RAMP2. n ≥ 4, Data were compared by Student’s *t* test, *p < 0.05, **p < 0.01, ***p < 0.001. Full length blots of panels A and C are shown in Supplementary Fig. 9.

To further examine the role of HRS in the degradation of CLR•RAMP2, we used two separate siRNAs to knockdown expression of HRS. Quantification of HRS knockdown was determined by Western blotting (Fig. 8A,B). HEK cells transiently expressing CLR•RAMP2 that had been treated with transfection reagent alone (Mock), non-targeting pool of siRNA (Scr) or two single siRNAs against HRS were exposed to AM (0–4 h) and degradation of CLR and RAMP2 examined by Western blotting. In Mock and Scr cells, both CLR and RAMP2 were degraded (Fig. 8A,C,D, Supplementary Fig. 11A,B). Surprisingly, knockdown of HRS had no effect of the degradation of CLR and RAMP2 (Fig. 8A,C,D).

### CLR•RAMP2 constitutively traffics to HRS-positive vesicles

**Figure 9:**
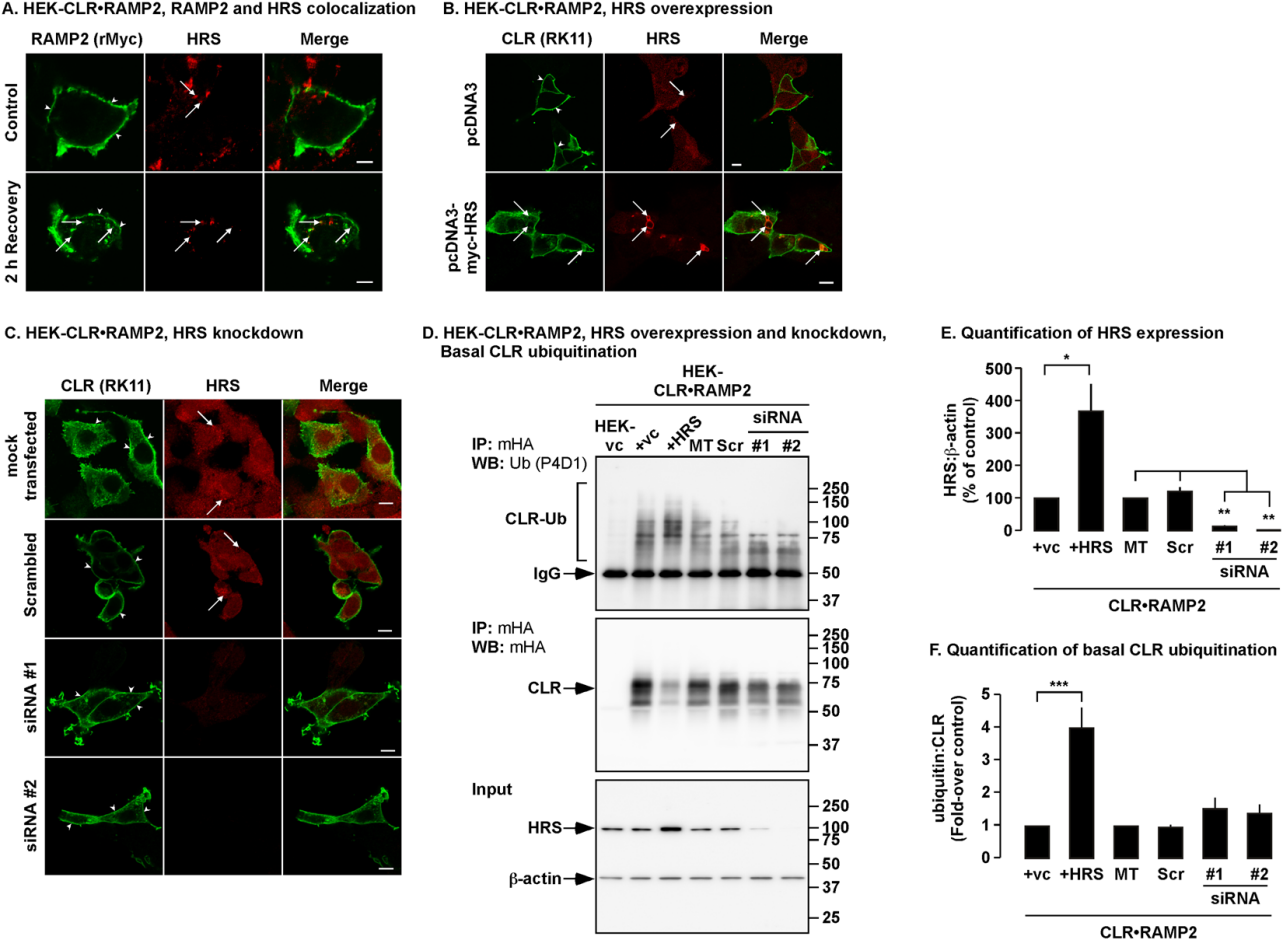
Colocalization of CLR and RAMP2 with HRS and effect of HRS expression on the basal levels of CLR ubiqutination. (**A**) HEK cells expressing CLR•RAMP2 were live labeled with an antibody to the extracellular epitope tag of RAMP2 (rabbit-Myc, rMyc) and fixed (control) or left for 2 h at 37 °C (2 h Recovery) before fixation. RAMP2 and HRS were localized using immunofluorescence. In control cells, RAMP2 was detected at the cell-surface (arrowheads). Following 2 h incubation, RAMP2 was also at the cell-surface (arrowheads), but could be detected in intracellular HRS-positive vesicles. (**B**) HEK cells expressing CLR•RAMP2 and empty vector (pcDNA3, control) or Myc-HRS were fixed permeablized and CLR (RK11) and HRS localized using immunofluorescence and confocal microscopy. In control cells CLR was detected at the cell-surface (arrowheads). In cells overexpressing HRS-myc, CLR was detected at the cell-surface, but also in enlarged HRS-positive endosomes. (**C**) HEK cells expressing CLR•RAMP2 and either mock transfected (control) or transfected a non-targeting pool siRNA (Scrambled) or siRNA to HRS (siRNA #1 and #2) were fixed and CLR and HRS localized by immunofluorescence and confocal microscopy. In all conditions CLR was detected at the cell-surface and HRS was detected in intracellular vesicles, with the exception of siRNA#1- and #2-treated cells were limited detection of HRS was possible due to knockdown. n = 3. Scale bar, 10 µm. (**D**) HEK cells expressing CLR•RAMP2 and either empty vector (pcDNA3, control, +vc), Myc-HRS, mock transfected (Mock, control) or transfected with a non-targeting pool siRNA (Scr) or siRNA to HRS (siRNA #1 and #2) were lysed, CLR immunoprecipitated (mouse HA, mHA) and Western blots probed using antibodies to ubiquitin and CLR (mHA). There were no signals for CLR in vector control cells, confirming antibody specificity. In Scr, siRNA#1 and siRNA#2 cells the basal levels of ubiquitination were similar to control (Mock). In contrast, in cells overexpressing HRS, the basal level of ubiquitinated CLR was significantly increased over control (+vc). (**E**) Quantification of HRS expression. (**F**) Quantification of ubiquitinated CLR. Data were examined using ANOVA and Student-Newman-Keuls post-hoc test, n = 4. *p < 0.05; **p < 0.01; ***p < 0.001. HEK-vc = HEK-vector control.

HRS regulates the ubiqutination and deubiquitination of the epidermal growth factor receptor^21^. We hypothesized that HRS may also regulate the degradation of CLR•RAMP2 by regulating the basal ubiquitination pattern of CLR. As a first step, we investigated if CLR•RAMP2 constitutively trafficked to HRS-positive vesicles. An antibody to the N-terminal epitope tag of RAMP2 was added to HEK cells expressing CLR•RAMP2 and cells immediately fixed or incubated for 2 h to allow constitutive trafficking to occur. We observed that CLR•RAMP2 constitutively internalizes and traffics to HRS-positive vesicles, presumably endosomes (Fig. 9A). Next, we determined the effect of HRS overexpression on the basal localization of CLR•RAMP2. In HEK-CLR•RAMP2 cells expressing empty vector (control), CLR was present at the cell-surface and HRS was present in intracellular vesicles (Fig. 9B). However, in HEK-CLR•RAMP2 cells overexpressing HRS, CLR was present at both the cell-surface and in HRS-positive intracellular vesicles (Fig. 9B). We also investigated the effect of HRS knockdown on the localization of CLR•RAMP2. We observed that for both siRNAs, CLR was present at the cell-surface, similar to that observed in Mock and Scr cells (Fig. 9C).

### HRS regulates the basal ubiquitination of CLR

As CLR was observed to co-localize with both endogenous and overexpressed HRS, we determined the effect of overexpressing and knockdown of HRS on the basal ubiquitination levels of CLR using immunoprecipitation and Western blotting. Quantification of HRS expression in each experimental condition was determined by Western blotting (Fig. 9D,E). In the control cells (empty vector, Mock, Scr) a comparable level of basal CLR ubiquitination could be detected (Fig. 9D,F). Similar basal levels of ubiquitination were also detected in cells lacking HRS (Fig. 9D,F). In contrast, basal CLR ubiquitination was significantly enhanced in cells overexpressing HRS (Fig. 9D,F). Therefore, CLR constitutively traffics to HRS-positive vesicles and HRS regulates the basal level of CLR ubiquitination.

### CLR•RAMP2 and CLRΔ9KR•RAMP2 traffic to HRS- and LAMP1-positive vesicles in HRS overexpressing cells

**Figure 10:**
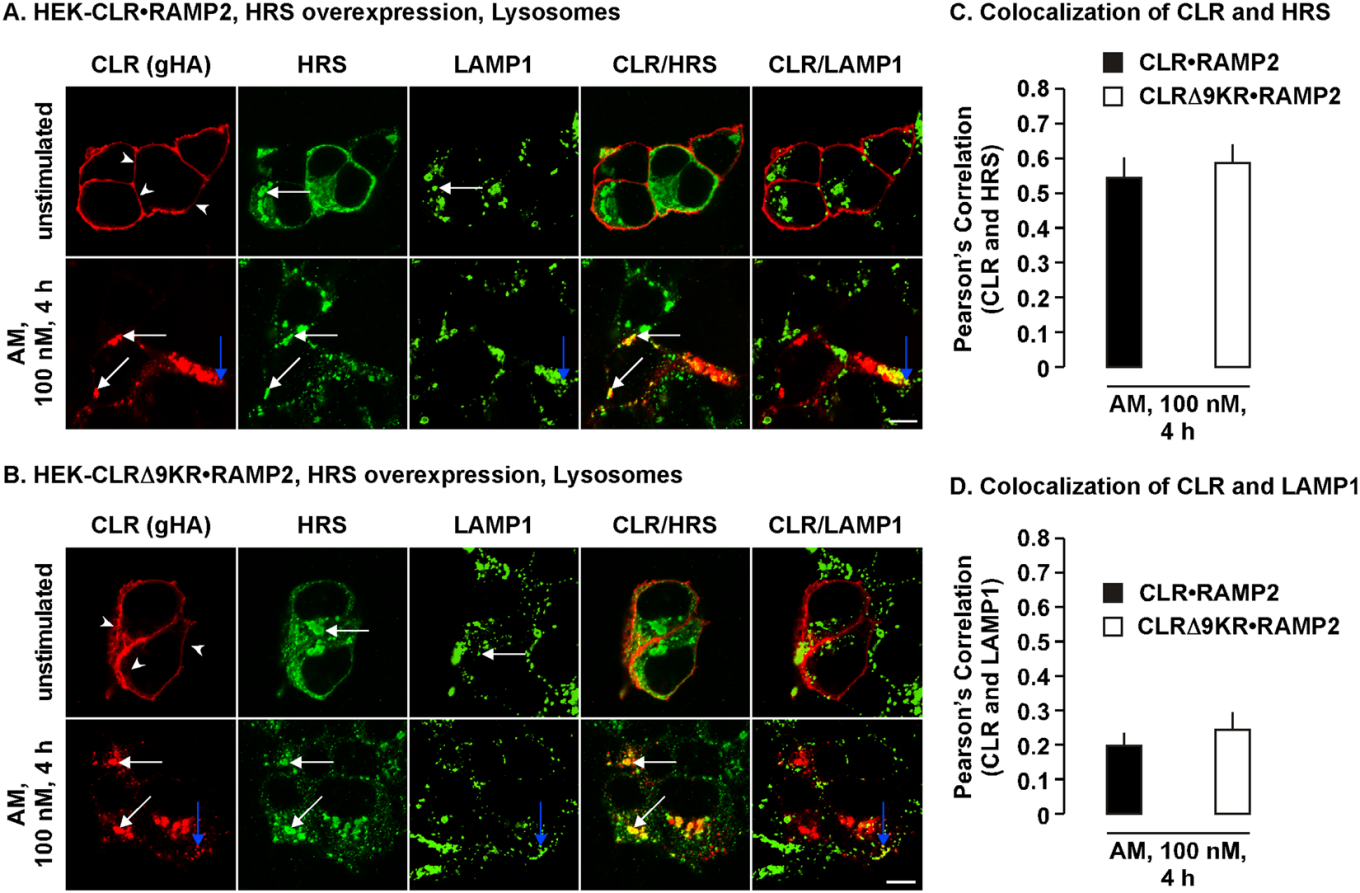
CLR•RAMP2 and CLRΔ9KR•RAMP2 traffic to HRS- and LAMP1-positive vesicles in HRS overexpressing cells. (**A,B**) In HEK-CLR•RAMP2 and HEK-CLRΔ9KR•RAMP2 cells overexpressing HRS, cell-surface CLR and CLRΔ9KR were labelled using an antibody to the epitope tag (goat-HA, gHA) were challenged with AM (100 nM, 0–4 h), fixed, permeabilized and CLR, CLRΔ9KR, HRS and a marker for lysosomes (LAMP1) localized by immunofluorescence and confocal microscopy. In unstimulated cells (top panels), CLR and CLRΔ9KR were present at the cell-surface (arrowheads) and LAMP1 was detected in intracellular vesicles (white arrows) and HRS in enlarged intracellular vesicles (white arrows). AM induced similar trafficking of CLR and CLRΔ9KR to colocalize with HRS (lower panels white arrows) and LAMP1 in lysosomes (blue arrows). (**C,D**) Quantification of CLR and CLRΔ9KR colocalization with HRS and LAMP1. n = 3. Data were examined using ANOVA and Student-Newman-Keuls post-hoc test. Scale bar, 10 µm.

Next we determined the effect of increased HRS expression on AM-induced trafficking of CLR and CLRΔ9KR. We overexpressed HRS, live labeled CLR at the cell-surface and used immunofluorescence and confocal microscopy to localize CLR, CLRΔ9KR, HRS and LAMP1 following treatment with AM (0–4 h). In unstimulated HEK-CLR•RAMP2 and HEK-CLRΔ9KR•RAMP2 cells, CLR was present at the cell-surface, HRS in enlarged endosomes and LAMP1 intracellular vesicles (Fig. 10A,B). Following stimulation with AM, CLR and CLRΔ9KR similarly trafficked to enlarged HRS-positive vesicles (Fig. 10A–C). However, both CLR and CLRΔ9KR could also be similarly detected in LAMP1-positive vesicles (Fig. 10A,C,D). Of note, we could also detect LAMP1 in HRS-positive vesicles (Supplementary Fig. S12A,B).

### Enhanced basal ubiquitination of CLR delays degradation of CLR•RAMP2

**Figure 11:**
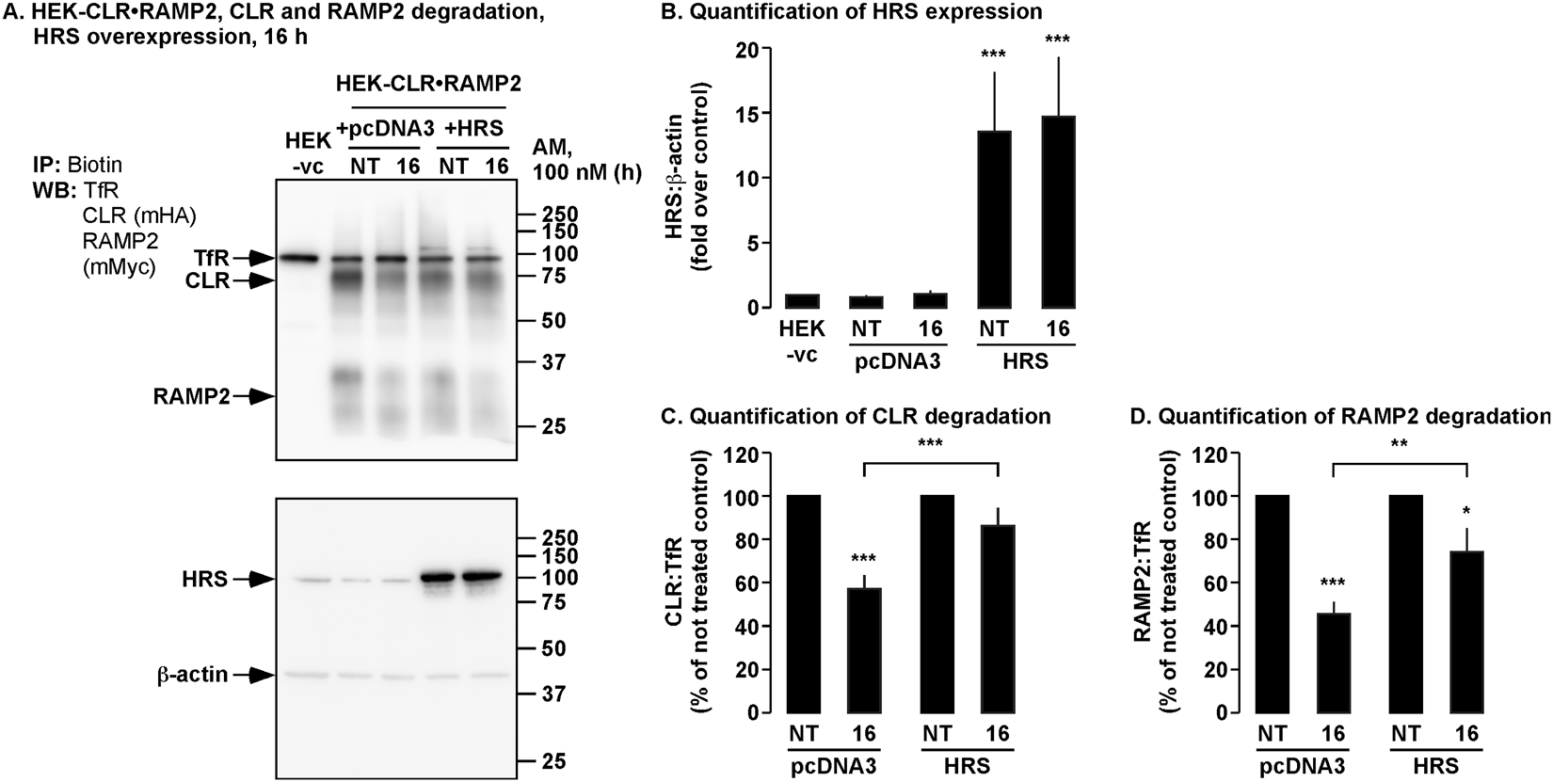
Effect of HRS overexpression on the degradation of CLR and RAMP2 following long term exposure to AM. (**A**) Cell-surface biotinylated HEK-CLR•RAMP2 cells expressing empty vector (control) or HRS were not treated (NT) or challenged with AM (100 nM, 16 h), biotinylated proteins immunoprecipitated (IP) and Western blots (WB) probed for CLR (mouse-HA, mHA), RAMP2 (mouse-Myc, mMyc) and transferrin receptor (TfR, loading control). In untreated cells, CLR, RAMP2 and TfR were readily detected. In control cells treated with AM (100 nM, 16 h) CLR and RAMP2 were significantly more degraded than in cells overexpressing HRS. Lysates from experimental cells were analyzed for the expression of HRS and β-actin. (**B**) Quantification of HRS expression. (**C,D**) Quantification of degradation of CLR and RAMP2. Data were examined using ANOVA and Student-Newman-Keuls post-hoc test. n = 4. *p < 0.05, **p < 0.01, ***p < 0.001. HEK-vc = HEK-vector control.

To investigate if overexpression of HRS simply ‘traps’ ubiquitinated CLR•RAMP2 in HRS-positive vesicles, we analyzed the degradation of CLR and RAMP2 following long-term exposure to AM (16 h). We first quantified expression of HRS in HEK cells expressing empty vector (control) or HRS and CLR•RAMP2 by Western blotting (Fig. 11A,B). We then treated the same cells with AM (0–16 h) and levels of CLR, RAMP2 and TfR quantified by Western blotting (Fig. 11A,C,D). In control cells, AM induced significant degradation of both CLR and RAMP2 compared to the not treated control and AM-treated cells overexpressing HRS. In contrast, AM induced significant degradation of RAMP2, but not CLR in cells overexpressing HRS (Fig. 11A,C,D). Thus, overexpression of HRS does not prevent, but only impedes the degradation of CLR•RAMP2.

## Discussion

In this study, we used HEK cells to detail the molecular mechanisms regulating the downregulation of the AM_1_ receptor. Our results show that AM induces trafficking of CLR•RAMP2 to early endosomes and then onto lysosomes. Furthermore, CLR is trafficked to lysosomes following both transient and sustained stimulation with AM. We also show that AM induces ubiquitination of CLR on intracellular lysines residues and that although ubiquitin does not target CLR•RAMP2 for degradation, a basal hyperubiquitination mediated by HRS overexpression does prevent degradation.

Ubiquitination has been shown to play a major role in the regulation of many GPCRs (reviewed in refs^43,44^). However, ubiquitination of CLR expressed with RAMP2 was unexpected, given that CGRP does not induce ubiquitination of CLR when expressed with RAMP1^14^. The kinetics of agonist-induced CLR ubiquitination was unexpected. Most previously reported agonist-induced ubiquitination of GPCRs occurs within minutes^28,30^. However, we only detected ubiquitinated CLR, 1 h post-stimulation. To our knowledge, the only other GPCR that undergoes delayed ubiquitination is the neurokinin 1 receptor. However, these studies were performed in a cell line that does not naturally express that receptor^45^. Our finding that AM induced ubiquitination of CLR in an epithelial (HEK) and endothelial cell (HMEC-1) suggests that this may be a universal mechanism that regulates AM_1_ receptors.

We generated a mutant CLR lacking all intracellular facing lysine residues to study the role of CLR ubiquitination. We observed that AM did not induce ubiquitination of CLRΔ9KR, but this lack of ubiquitination did not affect the endocytic trafficking to lysosomes. This finding was not unexpected as although ubiquitination does affect the trafficking of some GPCRs^28,30^, it does not regulate the lysosomal targeting of others^14,31,33^. We also observed a basal level of CLR ubiquitination, indicating that perhaps CLR is constitutively ubiquitinated. Again, this is not unusual as PAR1 is constitutively ubiquitinated in order to prevent its internalization^46^.

The duration of agonist exposure in known to influence ultimate fate of GPCRs. Transient exposure of CLR•RAMP1 to CGRP results in efficient recycling^14,15^. However, continued CGRP exposure promotes trafficking to lysosomes^14^. The trafficking of the neurokinin 1 receptor can be similarly modified^45,47^. CLR•RAMP3, the other receptor for AM, has been shown to internalise and recycle back to the cell-surface following agonist exposure^24^. Our results indicate that once activated CLR•RAMP2 is degraded. This was unexpected as we had expected CLR•RAMP2 to be similarly regulated by endothelin-converting enzyme 1, especially as this endosomal peptidase cleaves AM in a pH-dependent manner, similar to that observed with CGRP (Cottrell, unpublished observation). However, the lack of CLR•RAMP2 recycling is in agreement with a previous study where it has been reported that following a transient stimulation with AM (30 min), CLR•RAMP2 is internalised and less than 10% of CLR•RAMP2 recycles back to cell-surface after 2 h^25^. Thus, it appears that in addition to modifying the pharmacology of CLR^13^, RAMPs play an important role in the post-endocytic sorting of CLR following agonist stimulation and can change the fate of CLR•RAMP complex.

As CLR colocalized with the lysosomal marker LAMP-1, we expected that degradation of CLR•RAMP2 would be prevented by inhibitors of lysosomal peptidases. However, the lysosomal protease inhibitors cocktail that completely prevented CGRP-induced degradation of CLR•RAMP1^14^, did not affect AM-induced degradation of CLR•RAMP2. Furthermore, leupeptin that has been shown to prevent degradation of other GPCRs^41^, did not prevent degradation of the AM_1_ receptor. This result suggests that GPCRs may be degraded by different repertoires of peptidases. Studies have shown that δ-opioid receptor (δ-OR) degradation is prevented by MG-132, which is often used to inhibit the proteolytic activity of the proteasome^33,39^. However, MG-132 has been found to inhibit certain cathepsins e.g. B, L and K found in lysosomes^48–50^. Our data using the highly specific proteasome inhibitor, epoxomicin, suggests the proteasome has no role in the degradation of CLR•RAMP2.

Ubiquitin-dependent alterations in mitogenic signaling have been observed for other cell-surface receptors^36,38^. Ubiquitination of insulin-like growth factor 1 receptor is an absolute requirement for insulin-like growth factor-induced ERK activation^36^. We were unable to detect any changes in AM-induced mitogenic signaling, but that does not rule out the fact that other AM-induced signaling cascades may be altered.

Ubiquitination has been shown to participate in many different phases of GPCR regulation, such as promoting^27^ or preventing internalisation^46^, targeting to lysosomes for degradation^28,30^ and increasing the rate of degradation^31^. We examined if AM promoted degradation of CLR•RAMP2 and CLRΔ9KR•RAMP2 with different kinetics by examining degradation at early time points. However, we did not observe any significant differences in the degradation of CLR, CLRΔ9KR or RAMP2 in HEK cells or HMEC-1 cells. This finding was unexpected as ubiquitination usually either regulates internalization, lysosomal targeting or degradation kinetics^27,28,30,31,46^.

HRS overexpression regulates the degradation of the δ-OR^20^, protease-activated receptor 2, the CGRP receptor (CLR•RAMP1)^19^ and the recycling of the β_2_-adrenoceptor^18^. The pronounced difference in the sensitivity of CLR•RAMP2 and CLRΔ9KR•RAMP2 to HRS overexpression indicates that ubiquitination plays a key role in the movement of AM_1_ receptors through the endocytic machinery. However, as the lysine defective mutant is still degraded, an alternative pathway must be open to the receptor if it is not ubiquitinated. One possibility is that it could follow a similar pathway to PAR1 which has been shown to be degraded in lysosomes in an HRS- and ubiquitin-independent but ESCRT-III-dependent mechanism^51,52^, although both CLR and RAMP2 lacks the YPX(3)L motif necessary for the interaction with ALIX (an ESCRT-III–interacting protein). However, this still reinforces the idea that similar to the protease-activated receptors the AM_1_ receptor is a one-shot receptor because irrespective of whether the receptor is ubiquitinated or not, it is destined for degradation. Deletion of the UIM of HRS did not rescue CLR•RAMP2 degradation indicating HRS is not the endosomal sorting protein responsible for the recognition of ubiquitinated CLR. Numerous endosomal sorting proteins contain UIMs including EPS15, epsins, the ESCRT-0 component, STAM, the ESCRT-I component TSG101, the ESCRT-II component VPS36, as well as EPS15b and GGA3 (reviewed in ref.^53^). Thus, it is plausible that anyone of these proteins is responsible for the recognition and sorting of ubiquitinated AM_1_ receptors.

Considering that degradation of other GPCRs and their lysine-deficient mutants is inhibited by HRS knockdown^19,20^, it was surprising that knockdown of HRS had no effect on AM-induced degradation of CLR•RAMP2 or CLRΔ9KR•RAMP2. Furthermore, CLR•RAMP2 also localized to enlarged HRS-positive vesicles following HRS overexpression, similar to protease-activated receptor 2, CLR•RAMP1 and δ-OR^19,20^. Investigation into the effect of HRS overexpression on AM-induced trafficking revealed that both CLR and CLRΔ9KR accumulate in HRS- and LAMP1-positive vesicles, although it was clear that both receptors predominantly colocalized with HRS. It was surprising that there was no difference in the colocalization of CLR and CLRΔ9KR with LAMP1 and that the colocalization of CLRΔ9KR with LAMP1 was far less than in the absence of HRS overexpression given the HRS overexpression did not affect AM-induced degradation of CLRΔ9KR. It should also be noted that we used antibody-tagged receptors in our immunofluorescence experiments (to circumvent the high level of CLR colocalization with HRS under basal conditions) and so we visualized the antibodies used for tagging and not the receptors themselves. Therefore, we cannot rule out that the antibodies using for tagging the receptors are degraded at similar rates, whereas the receptors themselves are not. Furthermore, in our biochemical experiments, we cannot rule out pre-lysosomal proteolysis of the epitope tag used to detect both CLR and CLRΔ9KR.

Unlike other receptors that have been shown to be regulated by HRS overexpression it is clear from our results that HRS overexpression does not prevent degradation of CLR•RAMP2, but only delays degradation. Thus, it is possible that degradation of other GPCRs such as the δ-OR^20^ and PAR2^19^ is only retarded, as in those investigations longer time points (>4 h) were not investigated.

**Figure 12:**
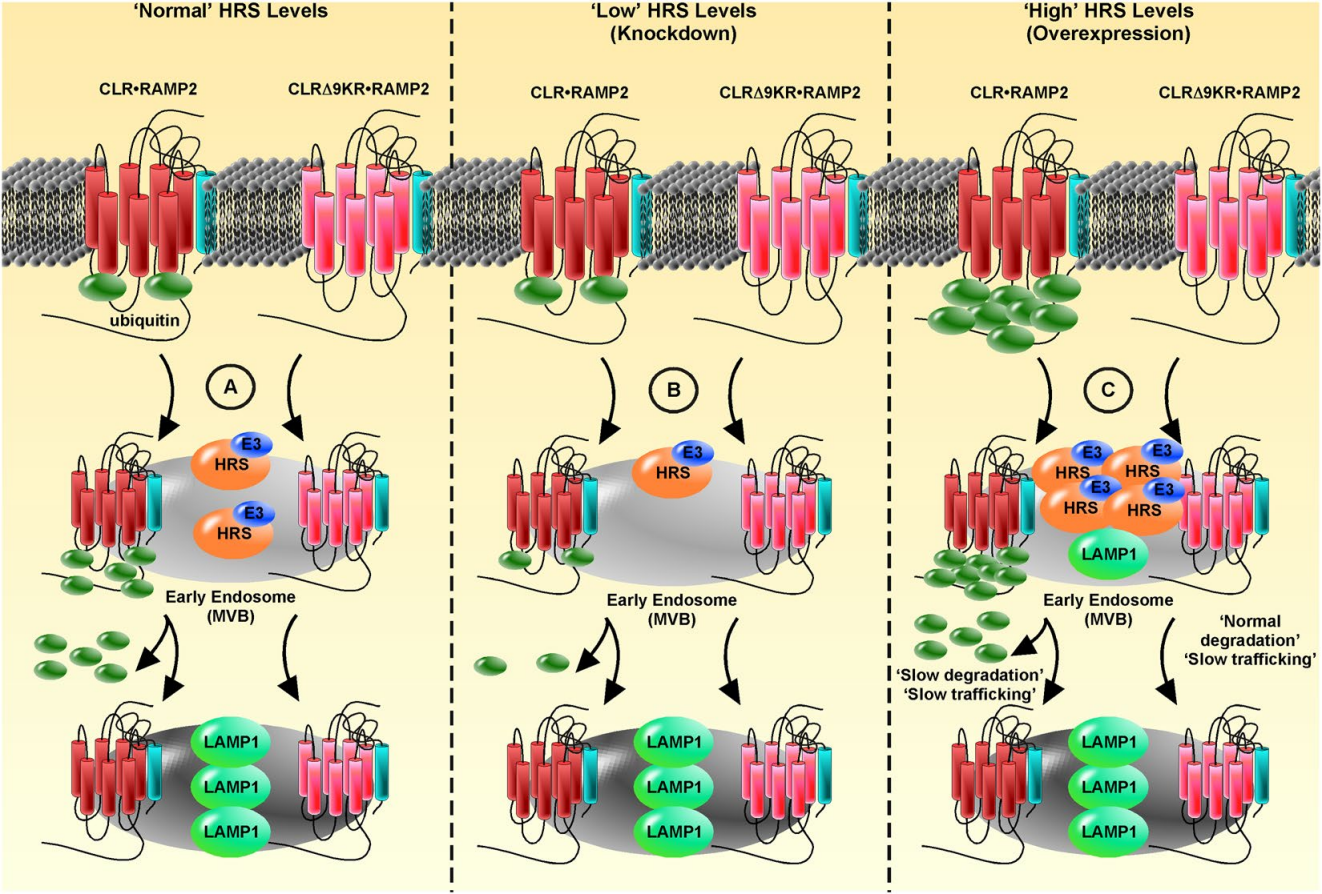
Proposed model for the regulation of CLR•RAMP2 by ubiquitination and HRS. (**A**) When HRS is expressed at endogenous levels, basal levels of CLR ubiquitination are normal and are regulated by an endosomal interaction with an HRS-linked E3 ligase during constitutive recycling. CLRΔ9KR is ubiquitin-free. Upon AM-induced activation both CLR•RAMP2 and CLRΔ9KR•RAMP2 internalize to endosomes where CLR•RAMP2 is ubiquitinated further. Both CLR•RAMP2 and CLRΔ9KR•RAMP2 traffic to the multi-vesicular body (MVB), where presumably CLR•RAMP2 is deubiquitinated. CLR•RAMP2 and CLRΔ9KR•RAMP2 are then delivered to LAMP1-positive vesicles. (**B**) Depleting HRS promotes a low basal level of ubiquitination of CLR•RAMP2 and CLRΔ9KR•RAMP2 is ubiquitin-free. Stimulation with AM promotes internalization of both CLR•RAMP2 and CLRΔ9KR•RAMP2 to the MVB, CLR may then be ubiquitinated and deubiquitinated. CLR•RAMP2 and CLRΔ9KR•RAMP2 are then delivered to LAMP1-positive vesicles. (**C**) If HRS is overexpressed the basal level of CLR ubiquitination is high due to increased recruitment of an E3 ligase to endosomes. AM-induced activation promotes trafficking of both CLR•RAMP2 and CLRΔ9KR•RAMP2 to the MVB. Hyperubiquitination retards the degradation and trafficking of CLR•RAMP2 to LAMP1-positive vesicles, whereas non-ubiquitinated CLRΔ9KR•RAMP2 is degraded as normal (possibly due to incorporation of LAMP1-positive vesicles into the MVB) by trafficking to LAMP1-positive vesicles is impeded.

Our discovery that CLR•RAMP2 constitutively traffics to HRS-positive endosomes and HRS overexpression promotes hyperubiquitination of CLR under basal conditions could provide an explanation for the differences observed in the degradation of CLR•RAMP2 and CLRΔ9KR•RAMP2 (Fig. 12). If HRS recruits an E3 ligase, such as UBE4B^21^ that ubiquitinates CLR, then the hyperubiquitination that occurs during the constitutive trafficking of CLR•RAMP2 could perturb the endocytic transport of CLR•RAMP2 to the lysosome following activation. A similar effect on the ubiquitination state and degradation of PAR2 is observed following overexpression of a dominant-negative mutant of the deubiquitinating enzyme, associated molecule with the SH3 domain of STAM (AMSH)^54^. In contrast, trafficking of non-ubiquitinated CLRΔ9KR•RAMP2 is compromised by disruption of the endocytic network by HRS overexpression, but degradation can still occur by incorporation of LAMP1-positive vesicles into the MVB. HRS knockdown on the other hand, would not result in increased ubiquitination and wild-type receptors would either traffic as normal or via the route of non-ubiquitinated mutant CLRΔ9KR•RAMP2. Our data shows that basal ubiquitination of CLR•RAMP2 can be regulated by HRS expression and that basal ubiquitination levels regulate agonist-induced degradation.

## Methods

### Reagents

Sources of antibodies were: rabbit anti-rat CLR (RK11) was a gift from Nigel W. Bunnett (Monash University, Victoria, Australia)^55^); mouse anti-pERK (E-4, raised to Tyr204, Lot No. L1311), rabbit anti-ERK2 (C-14, Lot No. A1509), mouse anti-ubiquitin (P4D1, Lot No. G0609), mouse anti-lysosomal-associated glycomembrane protein 1 (LAMP1, H4A3, Lot No. G0109, Insight Biotechnology, Wembley, UK); mouse anti-early endosomal antigen-1 (EEA1, BD Transduction Laboratories, Oxford, UK, Lot No. 52897); mouse anti-human transferrin receptor (Lot No. 136800), donkey anti-mouse or rabbit IgG coupled to AlexaFluor488 or 546 (Invitrogen, Paisley, UK); rabbit anti-HA (H6908, Lot No. 118K4800), mouse anti-β-actin (A5441, Lot No. 028K4826), rabbit anti-c-Myc (C3956, Lot No. 098K4806); mouse anti-c-Myc (clone 9E10, M4439, Lot No.) 121M4826; mouse anti-HRS (WH0009146M1, Lot No. E5221-6D11) and rabbit anti-HRS (HPA007728, Lot No. A104535) (Sigma-Aldrich Company Ltd., Dorset, UK); mouse anti-HA.11 (clone 16B12; Cambridge Bioscience, Cambridge, UK, Lot No. B204538); goat anti-HA.11 (GTX30545, Insight Biotechnology, Lot No. 821700278); rat anti-HA.11 (Roche, Burgess Hill UK, Lot No. 14559100); goat or donkey anti-mouse, rat or rabbit IgG coupled to horseradish peroxidase, fluorescein isothiocyanate, Rhodamine Red-X, DyLight 649 or Cy5 (Stratech Scientific Limited, Newmarket, UK); Rat AM (Bachem, Weil am Rhein, Germany). DharmaFECT 1 Transfection Reagent, ON-TARGETplus HRS siRNA (J-016835-05-0002, J-016835-06-0002) and ON-TARGETplus (D-001810-10-05) (GE Healthcare, Little Chalfont, UK). Other reagents were from Sigma-Aldrich Company Ltd unless stated.

### Plasmids

cDNA encoding rat CLR has been described^14,55^. cDNA encoding rat RAMP2 was obtained by RT-PCR from rat heart using Trizol^®^ (Invitrogen) and Taqman Reverse Transcription reagents (Applied Biosystems, Warrington, UK) according to the manufacturer’s guidelines. An N-terminal Myc tag was added to RAMP2 by PCR. A single vector (pcDNA5/FRT) expressing both rat CLR and RAMP2 was created as described for CLR and RAMP1^14^. A CLR mutant in which all predicted intracellular lysines were replaced with arginines (designated CLRΔ9KR) was generated using a mega-primer PCR method. Lentiviral vectors expressing either CLR or CLRΔ9KR and RAMP2 were generated by releasing expression cassettes from existing plasmids by restriction digest, followed by cloning into a circularized pLenti6.3V5 (Invitrogen). All PCR amplified constructs were sequenced to verify integrity. Primer sequences are available on request. pcDNA3.0-Myc-HRS and Myc-HRSΔUIM were a kind gift from Professor M. von Zastrow (University of California, San Francisco, CA).

### Transfected Cells and Cell Lines

Human embryonic kidney (HEK293) cells containing the Flp-In™ system (HEK-FLP) were from Invitrogen (Paisley, UK). Cells were cultured in Advanced Dulbecco’s modified Eagle’s medium (DMEM) supplemented with l-glutamine (2 mM), 2% heat-inactivated fetal bovine serum (FBS) and zeocin (100 μg/ml). HEK-FLP cells stably expressing CLR and RAMP2 from the same vector (pcDNA5/FRT) were created with the Flp-In™ system according to the manufacturer’s guidelines, and cells were grown in Advanced DMEM supplemented with l-glutamine (2 mM), 2% heat-inactivated FBS and hygromycin B (200 µg/ml). SV40 large T Ag-transformed human dermal microvascular endothelial cells (HMEC-1^56^) were obtained from Centre for Disease Control and Prevention (Atlanta, Georgia) and were grown in MCDB131 supplemented with 10% heat-inactivated FBS, l-glutamine (2 mM), hydrocortisone acetate (1 µM) and human epidermal growth factor (10 ng/ml). HEK293T cells were grown in DMEM containing 10% heat-inactivated fetal bovine serum. All cells were routinely grown in 95% air, 5% CO_2_ at 37 °C. In control experiments, cells were stably transfected with vectors without inserts (HEK-vector control, HEK-vc; HMEC-1-vector control, HMEC-1-vc). In experiments involving expression of HRS, cells were transiently transfected using polyethylenimine (ratio 3:1 w/w).

### Lentivirus Production and Transduction

HEK293T cells were plated (1 × 10^5^ cells/cm^2^ in 100 mm dishes) and transiently transfected in DMEM/HEPES (25 mM) containing 10% heat-inactivated fetal bovine serum with pLenti6.3-control, pLenti6.3-CLR•RAMP2, pLenti6.3- CLRΔ9KR•RAMP2 (9 µg) together with vectors required for viral competence (pMDG.1, VSV-G envelope, 4.5 µg; pRSV.rev, HIV-1 Rev, 2.25 µg; pMDLg/p.RRE, packaging plasmid, 4.5 µg) using a standard calcium phosphate method for 12 hours. The medium was then exchanged and collected and filtered (0.45 µm) 48 h later. Viral particles were then collected by centrifugation (40,000 *g*, 6 h, 4 °C) and resuspended in HMEC-1 medium (1 ml). HMEC-1 cells (2.6 × 10^4^ cells/cm^2^ in 6-well plates) were incubated (30 min, 4 °C) with viral suspensions including polybrene (5 µg/ml). Fresh medium (1 ml) was added and cells incubated (overnight, 37 °C). The following days cells were washed and placed HMEC-1 medium for a further 48 h. HMEC-1 cells were then passaged and placed in 100 mm dishes in HMEC-1 medium containing blasticidin (10 µg/ml). After two weeks resistant cells were pooled and cells expressing CLR at the cell-surface isolated using an antibody to the extracellular epitope tag (rat-HA) and Dynabeads (Invitrogen). Protein expression was verified by Western blotting and immunofluorescence and confocal microscopy.

### Activation of CLR•RAMP2 and Drug Treatments

48 h after plating cells or after transfection, cells were washed three times with PBS and placed in DMEM or MCDB131 containing 0.1% BSA (DMEM-BSA or MCDB131-BSA). Cells were stimulated with 100 nM rat AM for the indicated times and controls were left untreated for the duration of the experimental time course. Lysosomal proteases were inhibited using Z-Phe-Ala-diazomethylketone (Bachem, 200 µM), E64d and pepstatin A (Enzo Life Sciences, Exeter, UK; each 10 µM) or leupeptin (Roche, 10 µM). The proteolytic activity of the proteasome was inhibited using epoxomicin (1 µM, Enzo Life Sciences). Controls included appropriate vehicle and inhibitors were present throughout the experimental time courses and added to cells 30 min prior to stimulation.

### SDS-PAGE and Western Blotting

Whole cell lysates (10–30 µg protein) and immunoprecipitations were separated by SDS-PAGE (8, 12 or 15% acrylamide). Proteins were transferred to PVDF membranes (Immobilon-P, Millipore) and blocked for 1 h at room temperature (1x PBS, 0.1% Tween^20^, 2% BSA or 5% milk powder). Membranes were incubated with antibodies to pERK (1:5000), ERK2 (1:10,000), rabbit or mouse c-Myc (1:5-10,000), rabbit or mouse HA (1:10,000), ubiquitin (1:5000), anti-mouse HRS (1:1000), β-actin (1:100,000) or human transferrin receptor (1:10,000) (overnight, 4 °C; 1x PBS, 0.1% Tween^20^, 2% BSA or 5% milk powder). Membranes were washed for 30 min (1x PBS, 0.1% Tween^20^) and incubated with appropriate secondary antibodies coupled to horseradish peroxidase (1:10,000, 1 h, room temperature). Immunoreactive proteins were detected using enhanced chemiluminescence (BioRad or Geneflow Ltd.). Densitometric analysis was performed using ImageJ software or using an ImageQuant-RT ECL imaging system (GE Healthcare) and analyzed using ImageQuant TL software.

### Cell-Surface Biotinylation Assays

To biotinylate cell surface proteins cells were washed in 100 mM PBS, pH 7.4, and incubated with 0.3 mg/ml EZ-Link^™^-Sulfo-NHS-Biotin (Pierce) in PBS (30 min, 4 °C). Cells were washed in PBS, stimulated with AM (100 nM, 0-16 h in DMEM-BSA or MCDB131-BSA), lysed in RIPA buffer (50 mM Tris/HCl, pH 7.4, 150 mM NaCl, 5 mM MgCl_2_, 1 mM EGTA, 10 mM NaF, 10 mM Na_4_P_2_O_7_, 0.1 mM Na_3_VO_4_, 0.5% Nonidet P-40, peptidase inhibitor cocktail (Roche)), and centrifuged. Biotinylated proteins were recovered by incubation with NeutrAvidin-agarose (30 µl, overnight, 4 °C), pelleted, washed with RIPA buffer, boiled in Laemmli buffer, and analyzed by Western blotting.

### RNA interference

HEK293 cells were plated in 12-well plates (1.2 × 10^5^ cells/well) and left for 24 h, before siRNA transfections. Cells were incubated with vehicle or siRNA (25 nM)/transfection reagent complexes (DharmaFECT, 5 µl) for 24 h before transfection with CLR•RAMP2 using Lipofectamine2000 according to the manufacturer’s guidelines. Cells were used for experimentation after a further 48 h.

### Immunoprecipitation

Cells were lysed in RIPA (including N-ethylmaleimide, 10 mM for HMEC-1 immunoprecipitations) and centrifuged (10,000 *g*, 20 min, 4 °C). Supernatants were rotated with immunoprecipitating antibody (CLR or CLRΔ9KR, 0.5 µl; rabbit c-Myc, 3 µg; rat anti-HA, 1 µg; overnight, 4 °C). Protein A/G PLUS (Insight Biotechnology; 30 μl) was added and samples were rotated (2 h, 4 °C). Immunoprecipitates were pelleted, washed with RIPA buffer, boiled in Laemmli buffer and analyzed by SDS-PAGE and Western blotting.

### Immunofluorescence and Confocal Microscopy

Following drug treatments cells were washed in 100 mm PBS, pH 7.4, and fixed in PBS containing 4% paraformaldehyde, pH 7.4 (20 min, 4 °C). Cells were washed with PBS containing 0.1% saponin and 2% normal horse serum for 30 min. Proteins were localized using the primary antibodies CLR (RK11, 1:2000), EEA1 (1:500), LAMP1 (1:1000), rabbit or mouse anti-HRS (1:100) (overnight, 4 °C). Cells were washed and incubated with secondary antibodies coupled to fluorescein isothiocyanate, rhodamine Red-X, or Cy5 (1:500, 2 h, room temperature). To examine trafficking of CLR•RAMP2 from the cell-surface, CLR, CLRΔ9KR and RAMP2 were labeled by incubating cells with antibodies to the extracellular epitope tags (CLR, rat anti-HA, 1:1000 or goat anti-HA.11, 1:100; RAMP2, rabbit anti-c-Myc, 1:1000; 30 min, 37 °C). Cells were washed with PBS, stimulated with AM, fixed and incubated with appropriate secondary antibodies. Cells were observed with a Zeiss laser-scanning confocal microscope (LSM Meta 510 or LSM 510) using a Plan-Apochromat 63x/1.4 Oil DIC objective. Alternatively, cells were observed with a Nikon Eclipse Ti laser-scanning confocal microscope using a 100x/1.45 Oil DIC N2 objective. Images were collected at a zoom of 1-2 and an iris of <3 μm, and at least five optical sections were taken at intervals of 0.5 μm. Single sections are shown. Images were processed using ImageJ and Adobe Photoshop software. Colocalization of proteins was determined using the NIS-Elements AR software.

### Statistics

Results are expressed as mean ± S.E.M. of *n* ≥ 3 experiments and were compared by Student’s *t* test. Differences among multiple groups were examined using ANOVA and Student-Newman-Keuls post-hoc test. *p < 0.05, **p < 0.01, ***p < 0.001 were considered to be significant. Immunofluorescence images and blots represent *n* ≥ 3 experiments.

## Electronic supplementary material

Supplementary Information
