# Supplementary material for: The Role of Ubiquitination and Hepatocyte Growth Factor-Regulated Tyrosine Kinase Substrate in the Degradation of the Adrenomedullin Type I Receptor
Source: Sci Rep. 2017 Sep 28;7:12389. doi: 10.1038/s41598-017-12585-z (PMC5620052; doi:10.1038/s41598-017-12585-z)
Supplement: Supplementary file 1 — Supplementary Information [file 41598_2017_12585_MOESM1_ESM.doc]

**The Role of Ubiquitination and Hepatocyte Growth Factor-Regulated Tyrosine Kinase Substrate in the Degradation of the Adrenomedullin Type I Receptor**

**Benoît T. Roux^2^, Claudia C. Bauer^1^, Alister J. McNeish^1^ Stephen G. Ward^2^, and Graeme S. Cottrell^1^***

^1^Cellular and Molecular Neuroscience, Reading School of Pharmacy, University of Reading, RG6 6UB. UK

^2^Department of Pharmacy and Pharmacology, University of Bath, Claverton Down, Bath, BA2 7AY. UK

*corresponding author: [g.s.cottrell@reading.ac.uk](mailto:g.s.cottrell@reading.ac.uk)

**Supplemental Information**

**Supplemental Figures and Legends**


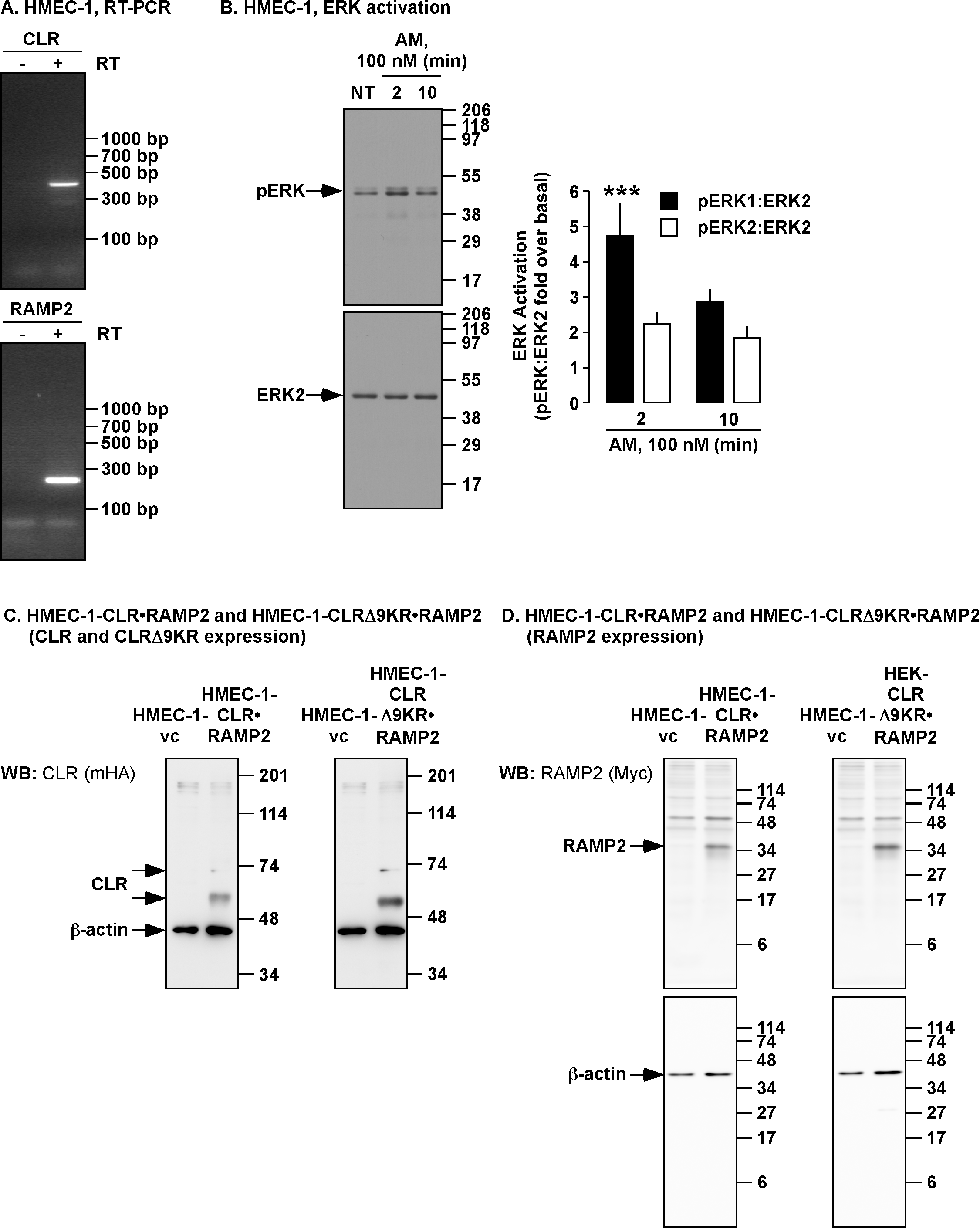


**Figure S1.** Expression of endogenous and exogenous CLR and RAMP2 in HMEC-1 cells. **(A**) RT-PCR amplification of mRNA in HMEC-1 cells encoding CLR (401 bp) and RAMP2 (267 bp). RT=reverse transcriptase. (**B**) Serum-starved HMEC-1 cells not treated (NT) or incubated with AM (100 nM, 0-10 min). Lysates were then analyzed for levels of phosphorylated ERK1/2 (pERK1/2) and ERK2 by Western blotting. In untreated cells, levels of pERK1/2 were low and AM-induced an increase in levels of pERK1/2 (pERK1; 2 min, 4.7±0.9; 10 min, 2.8±0.4; pERK2, 2 min, 2.2±0.3; 10 min, 1.8±0.3 fold over basal). (**C, D**) Lysates from HMEC-1-CLR•RAMP2 and HMEC-1-CLRΔ9KR•RAMP2 cells were analyzed by Western blot (WB) and probed for antibodies to the extracellular epitope tags of CLR (mouse-HA, mHA), CLRΔ9KR (mHA), RAMP2 (Myc) and β-actin (loading control). There were no signals for CLR, CLRΔ9KR and RAMP2 in HEK-vector control (HEK-vc) cells confirming specificity of detection. HA and Myc antibodies detected signals for CLR and CLRΔ9KR (~57 and 78 kDa) and RAMP2 (~37 kDa). Signals for β-actin were readily detected in all lysates. n=3-4. ***p<0.001.

**
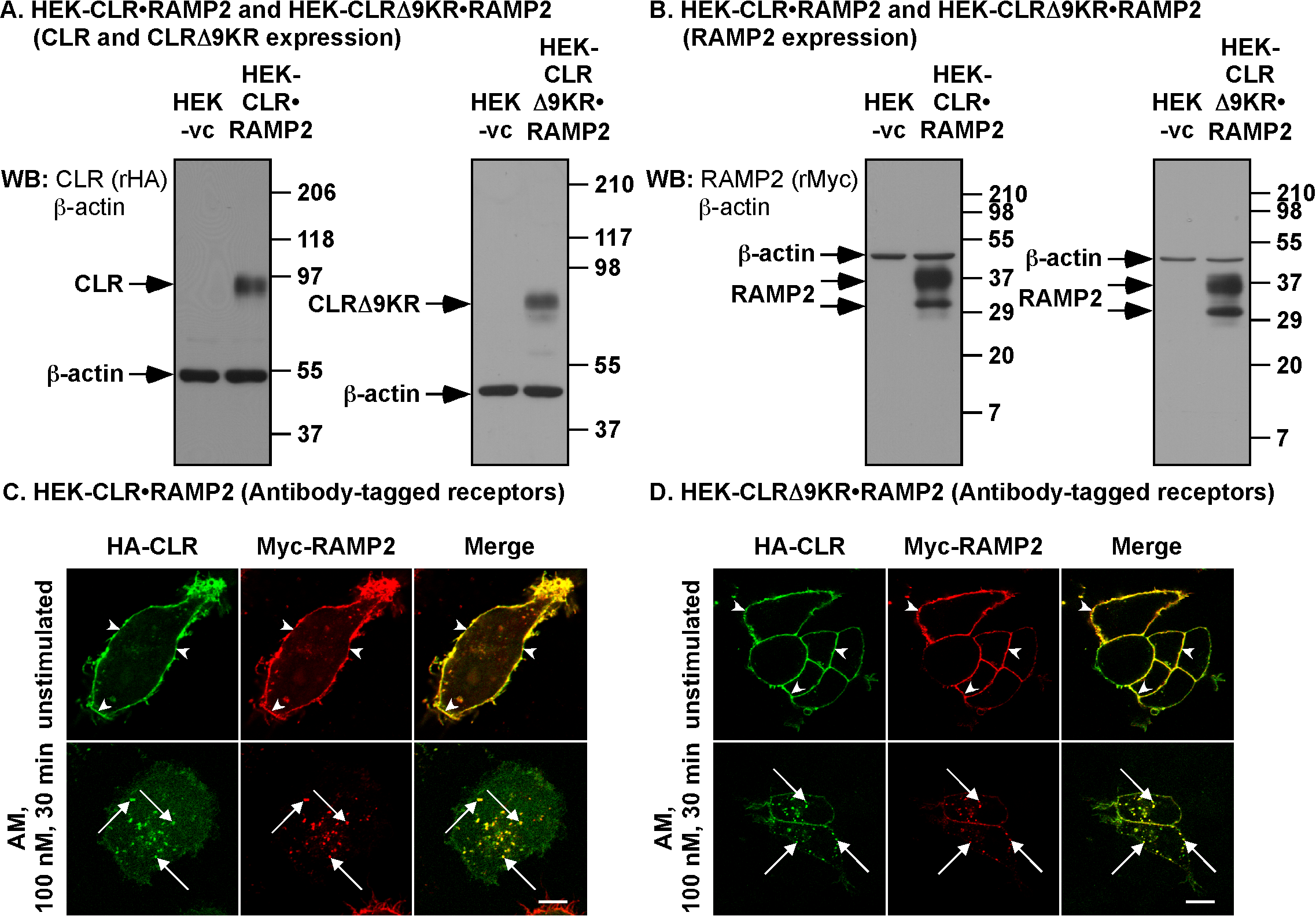
**

**Figure S2.** CLRΔ9KR•RAMP2 is expressed and traffics similarly to CLR•RAMP2. (**A, B**) HEK-CLR•RAMP2 and HEK-CLRΔ9KR•RAMP2 lysates were analyzed by Western blot (WB) and probed with antibodies to the epitope tags of CLR (rabbit-HA, rHA), CLRΔ9KR (rHA), RAMP2 (rabbit-Myc, rMyc) and β-actin (loading control). There were no signals in HEK-vector control cells confirming specificity of detection. HA and Myc antibodies detected signals for CLR and CLRΔ9KR (~85 kDa) and RAMP2 (~32 and 37 kDa). Signals for β-actin were readily detected in all lysates. (**C, D**) HEK-CLR•RAMP2 and HEK-CLRΔ9KR•RAMP2 cells were labeled with antibodies to CLR (rat-HA), CLRΔ9KR (rat-HA), RAMP2 (rMyc) and exposed to vehicle (control) or AM (100 nM, 30 min). After fixation, cells were incubated with fluorescent secondary antibodies to detect CLR, CLRΔ9KR and RAMP2. In unstimulated cells, CLR, CLRΔ9KR and RAMP2 were at the cell-surface (arrowheads). AM induced endocytosis of CLR, CLRΔ9KR and RAMP2 into the same intracellular vesicles (arrows). Scale bar, 10 µm.

**
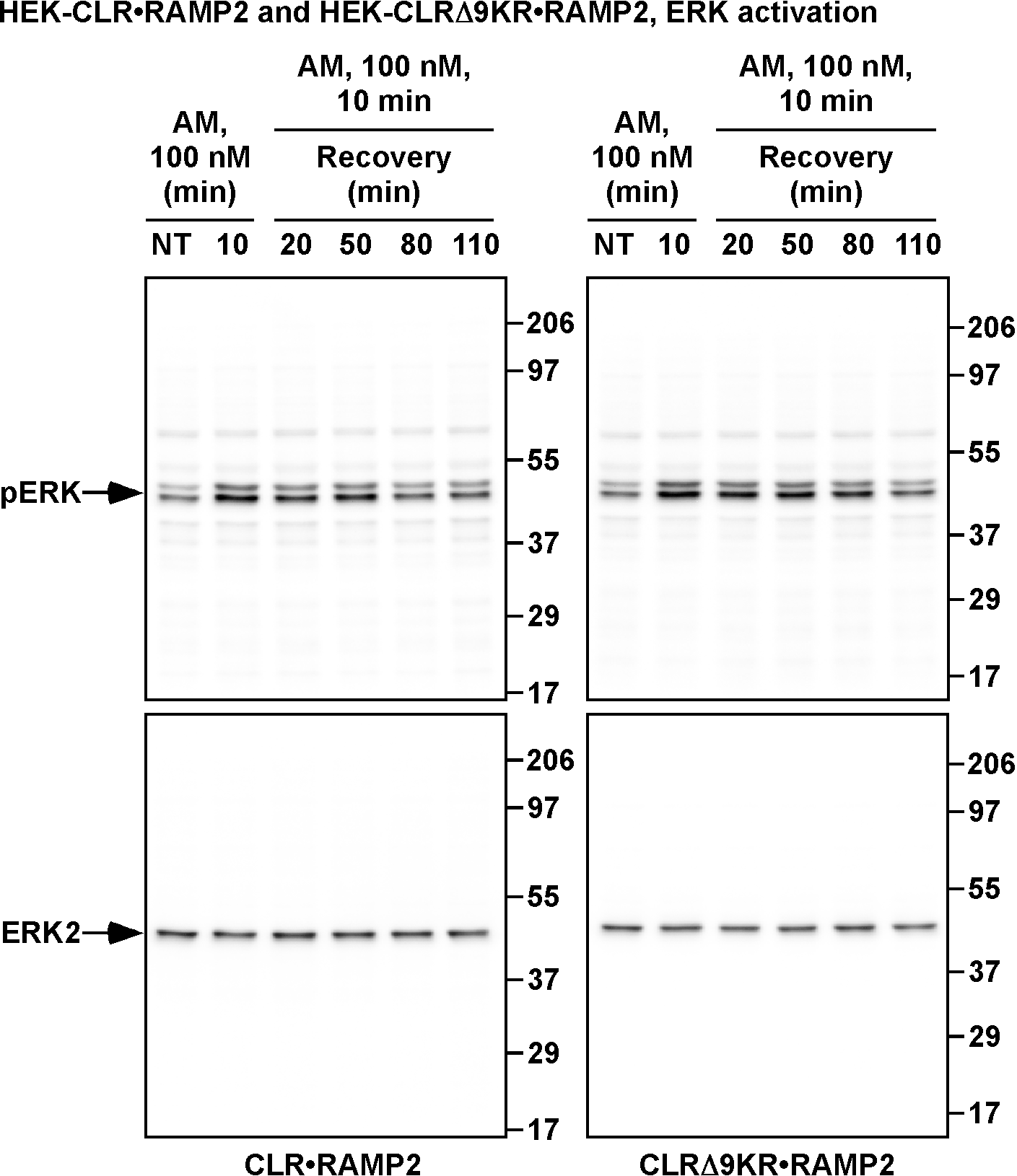
**

**Figure S3.** Serum-starved HEK-CLR•RAMP2 and HEK-CLRΔ9KR•RAMP2 cells were not treated (NT) or incubated with AM (100 nM, 0-10 min), washed and incubated in AM-free medium (0-120 min). Lysates were then analyzed for levels of phosphorylated (p) ERK2 (pERK2) and ERK2 by Western blotting. In untreated cells, levels of pERK2 were similarly low in both HEK-CLR•RAMP2 and HEK-CLRΔ9KR•RAMP2 cells. AM-induced a prompt increase in levels of pERK2 and returned towards basal levels after removal of agonist. The magnitude and duration of ERK2 activation is unaffected by the ubiquitination of CLR. This figure shows the full length blots in Fig. 2 of the manuscript.


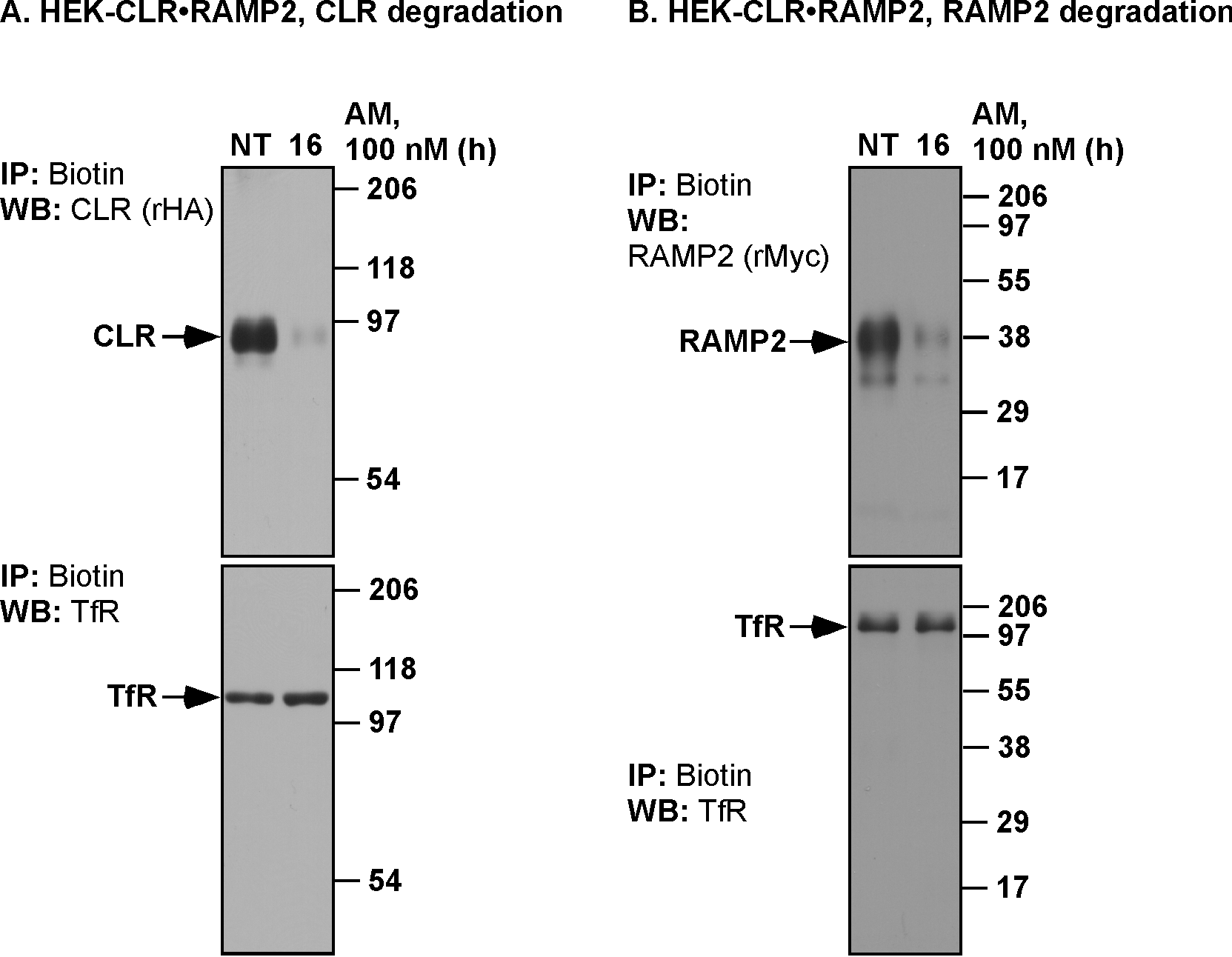


**Figure S4.** AM induces degradation of CLR and RAMP2 following continuous challenge. Cell-surface biotinylated HEK-CLR•RAMP2 were not treated (NT) or challenged with AM (100 nM, 16 h), biotinylated proteins immunoprecipitated (IP) and Western blots (WB) probed for CLR (rabbit-HA, rHA), RAMP2 (rMyc) and transferrin receptor (TfR, loading control). (**A, B**) In untreated HEK-CLR•RAMP2 cells, CLR, RAMP2 and TfR were readily detected. AM (100 nM, 16 h) induced degradation of CLR and RAMP2 to similar levels. This figure shows the full length blots in Fig. 4 (panels A and C) of the manuscript.

**
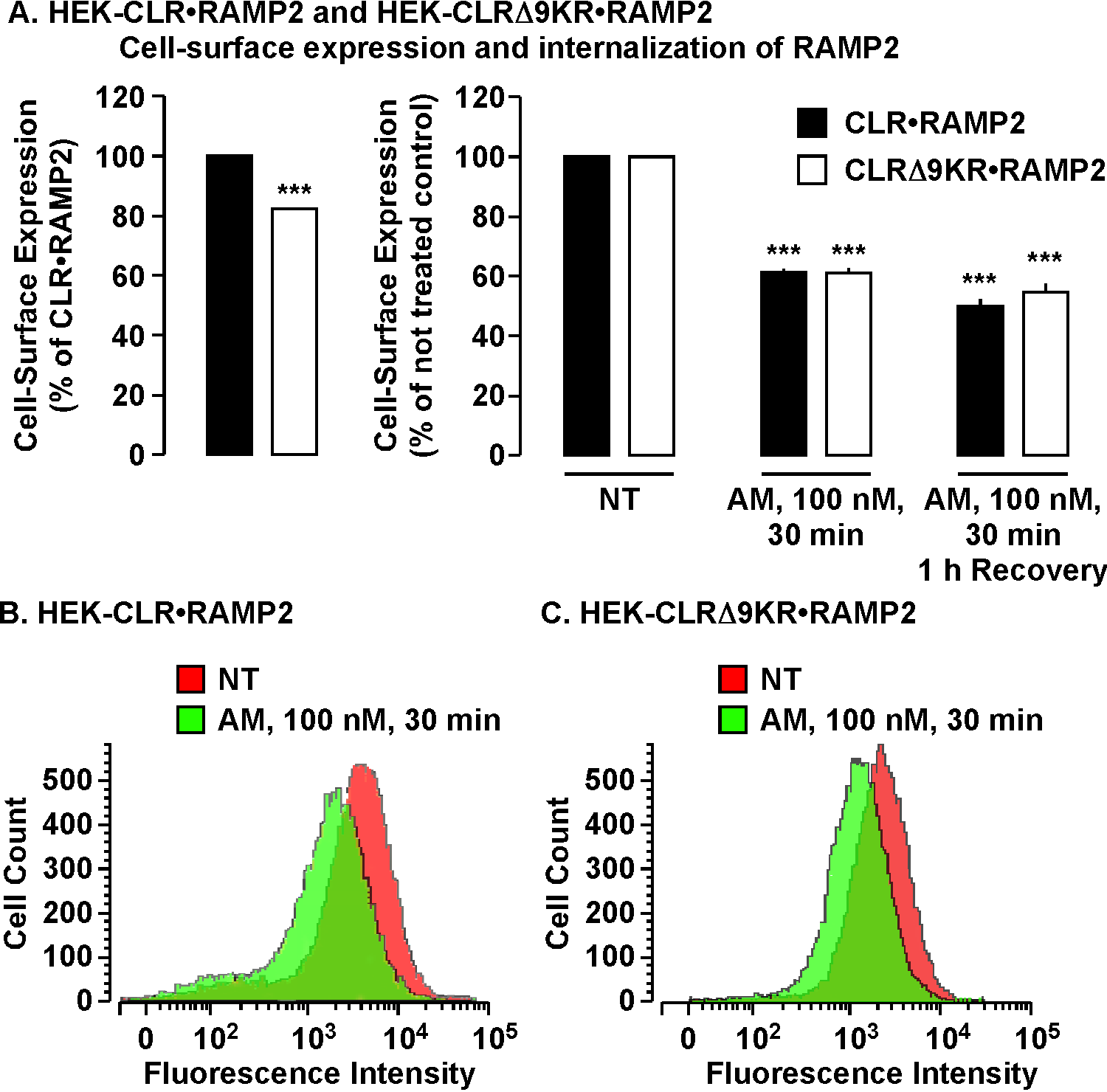
**

**Figure S5.** Quantification of cell-surface expression and internalization of CLR•RAMP2 and CLRΔ9KR•RAMP2. (**A, left panel**) Cell-surface expression of CLR•RAMP2 and CLRΔ9KR•RAMP2 was quantified by flow cytometry using an antibody to the extracellular epitope tag of RAMP2 (rabbit-Myc) (CLRΔ9KR•RAMP2, 82±0.4% compared to CLR•RAMP2 [100%]). (**A, right panel**) HEK-CLR•RAMP2 and HEK-CLRΔ9KR•RAMP2 cells were not treated (NT) or stimulated with AM (as described) and receptor internalization was quantified by flow cytometry. AM induced internalization of both CLR•RAMP2 and CLRΔ9KR•RAMP2 to similar levels. (**B, C**) Representative flow cytometry graphs showing internalization of CLR•RAMP2 and CLRΔ9KR•RAMP2 before and following stimulation with AM (100 nM, 30 min). n=3-4. Data were compared by Student's t test and differences among multiple groups were examined using ANOVA and Student-Newman-Keuls post-hoc test, ****p*<0.001 compared to CLR•RAMP2 (**A, left panel**) or NT control (**A, right panel**).

**
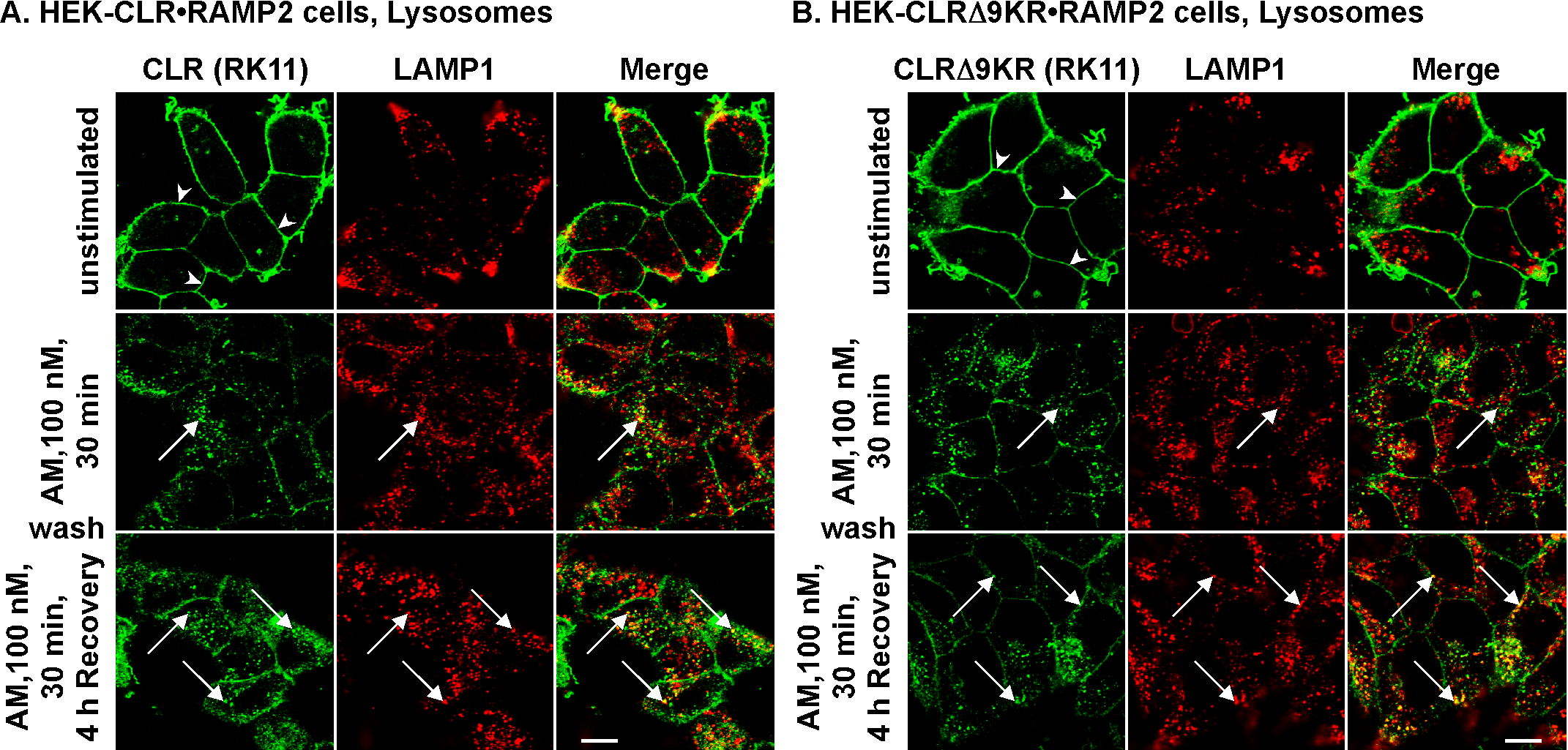
**

**Figure S6.** CLR and CLRΔ9KR•RAMP2 traffic to lysosomes following transient stimulation with AM. (**A, B**) HEK-CLR•RAMP2 and HEK-CLRΔ9KR•RAMP2 cells were left unstimulated or challenged with AM (100 nM, 30 min), washed and then incubated in AM-free medium for 4 h, fixed, permeabilized and CLR and a marker for lysosomes (LAMP1) localized by immunofluorescence and confocal microscopy. In unstimulated cells, CLR and CLRΔ9KR were present at the cell-surface (arrowheads) and LAMP1 was detected in intracellular vesicles. AM induced trafficking of CLR and CLRΔ9KR to colocalize with LAMP1 in lysosomes (arrows). n=3, Scale bar, 10 µm.

**
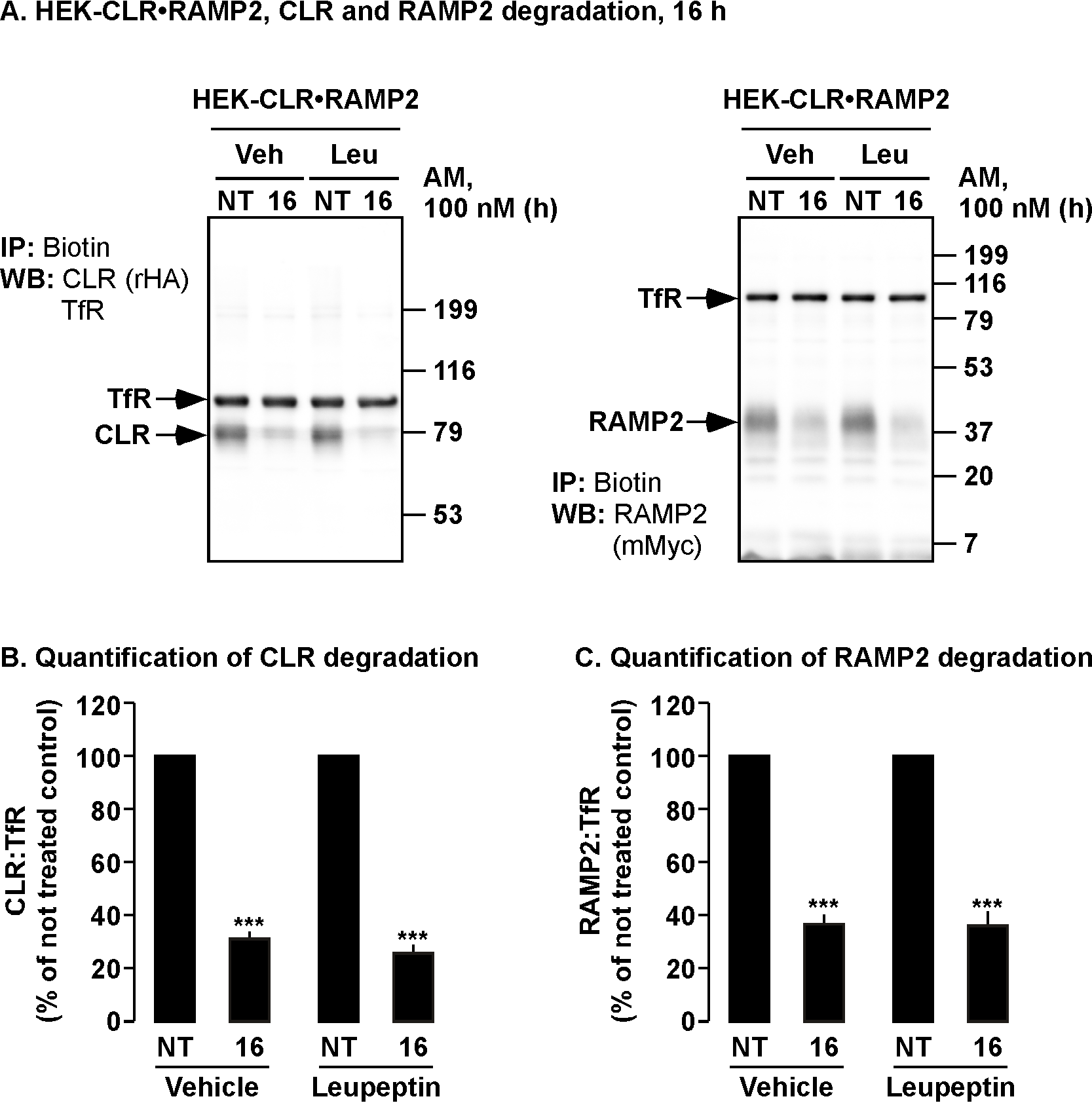
**

**Figure S7.** Effect of leupeptin on the degradation of CLR, CLRΔ9KR and RAMP2. (**A**) Cell-surface biotinylated HEK-CLR•RAMP2 cells were incubated with vehicle (control) or leupeptin (leu), not treated (NT) or challenged with AM (100 nM, 16 h), biotinylated proteins immunoprecipitated (IP) and Western blots (WB) probed for CLR (rabbit-HA, rHA), RAMP2 (mouse-Myc, mMyc) and transferrin receptor (TfR, loading control). In untreated HEK-CLR•RAMP2 cells, CLR, RAMP2 and TfR were readily detected. AM (100 nM, 16 h) induced degradation of CLR and RAMP2 to similar levels. (**B, C**) Quantification of the degradation of CLR and RAMP2. n=4. Data were examined using ANOVA and Student-Newman-Keuls post-hoc test. ****p*<0.001 compared to NT control.

**
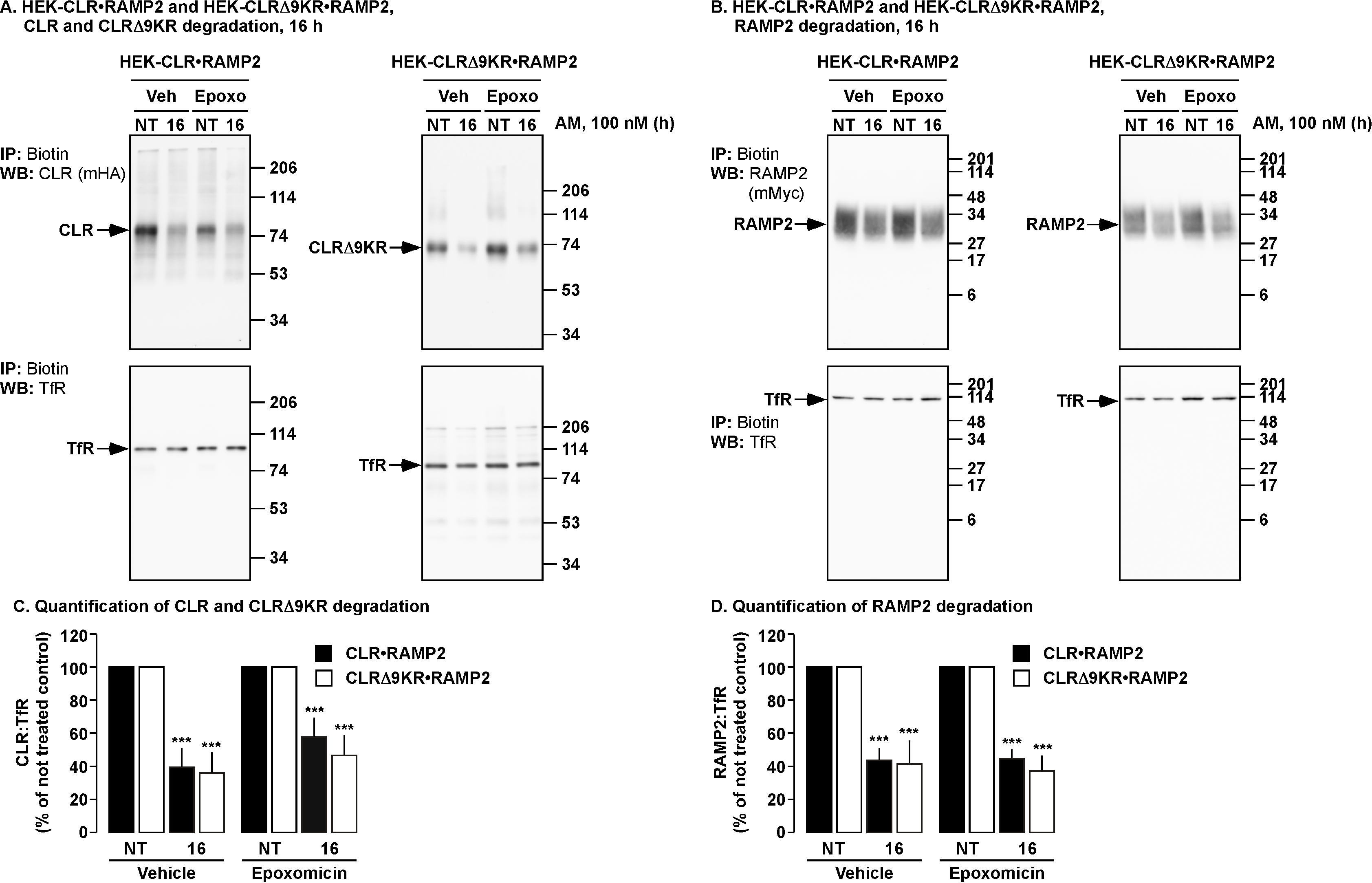
**

**Figure S8.** Effect of epoxomicin on the degradation of CLR, CLRΔ9KR and RAMP2. (**A, B**) Cell-surface biotinylated HEK-CLR•RAMP2 and HEK-CLRΔ9KR•RAMP2 cells were incubated with vehicle (control) or epoxomicin (epoxo), not treated (NT) or challenged with AM (100 nM, 16 h), biotinylated proteins immunoprecipitated (IP) and Western blots (WB) probed for CLR (mouse-HA, mHA), CLRΔ9KR (mHA), RAMP2 (mouse-Myc, mMyc) and transferrin receptor (TfR, loading control). In untreated HEK-CLR•RAMP2 and HEK-CLRΔ9KR•RAMP2 cells, CLR, CLRΔ9KR, RAMP2 and TfR were readily detected. AM (100 nM, 16 h) induced degradation of CLR, CLRΔ9KR and RAMP2 to similar levels. (**C, D**) Quantification of the degradation of CLR, CLRΔ9KR and RAMP2. n=4. Data were examined using ANOVA and Student-Newman-Keuls post-hoc test. ****p*<0.001 compared to NT control.

**
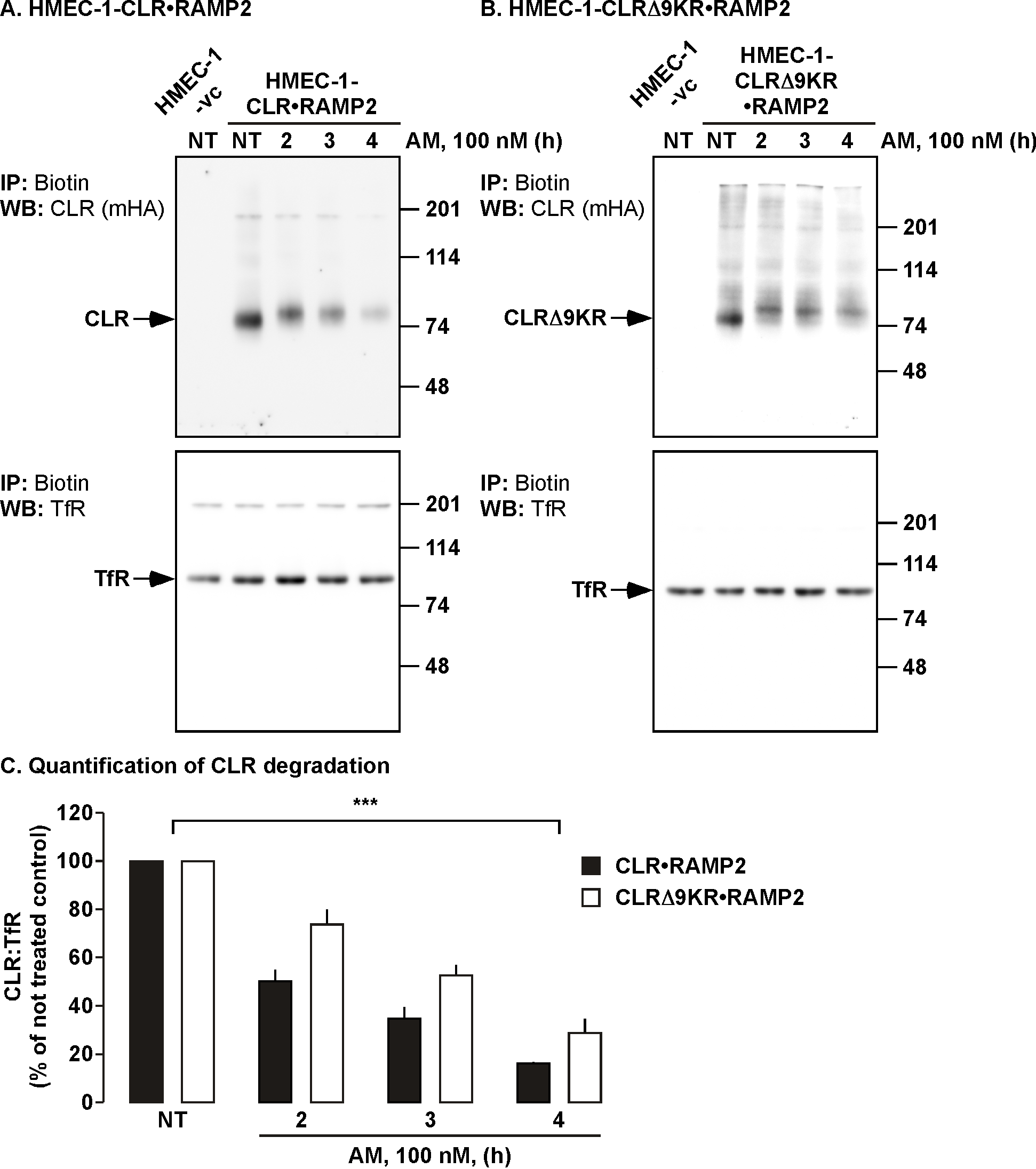
**

**Figure S9.** Degradation of CLR and CLRΔ9KR in HMEC-1 cells. Cell-surface biotinylated HMEC-1-CLR•RAMP2 and HMEC-1-CLRΔ9KR•RAMP2 cells were not treated (NT) or challenged with AM (100 nM, 4 h), biotinylated proteins immunoprecipitated (IP) and Western blots (WB) probed for CLR (mouse-HA, mHA), CLRΔ9KR (mHA) and transferrin receptor (TfR, loading control). In untreated HMEC-1-CLR•RAMP2 and HMEC-1-CLRΔ9KR•RAMP2 cells, CLR, CLRΔ9KR and TfR were readily detected. AM (100 nM, 4 h) induced similar rates of degradation of CLR and CLRΔ9KR. (**C**) Quantification of the degradation of CLR and CLRΔ9KR, respectively. n=4. Data were examined using ANOVA and Student-Newman-Keuls post-hoc test. ****p*<0.001 as compared to NT control. HMEC-1-vc=HMEC-1-vector control.

**
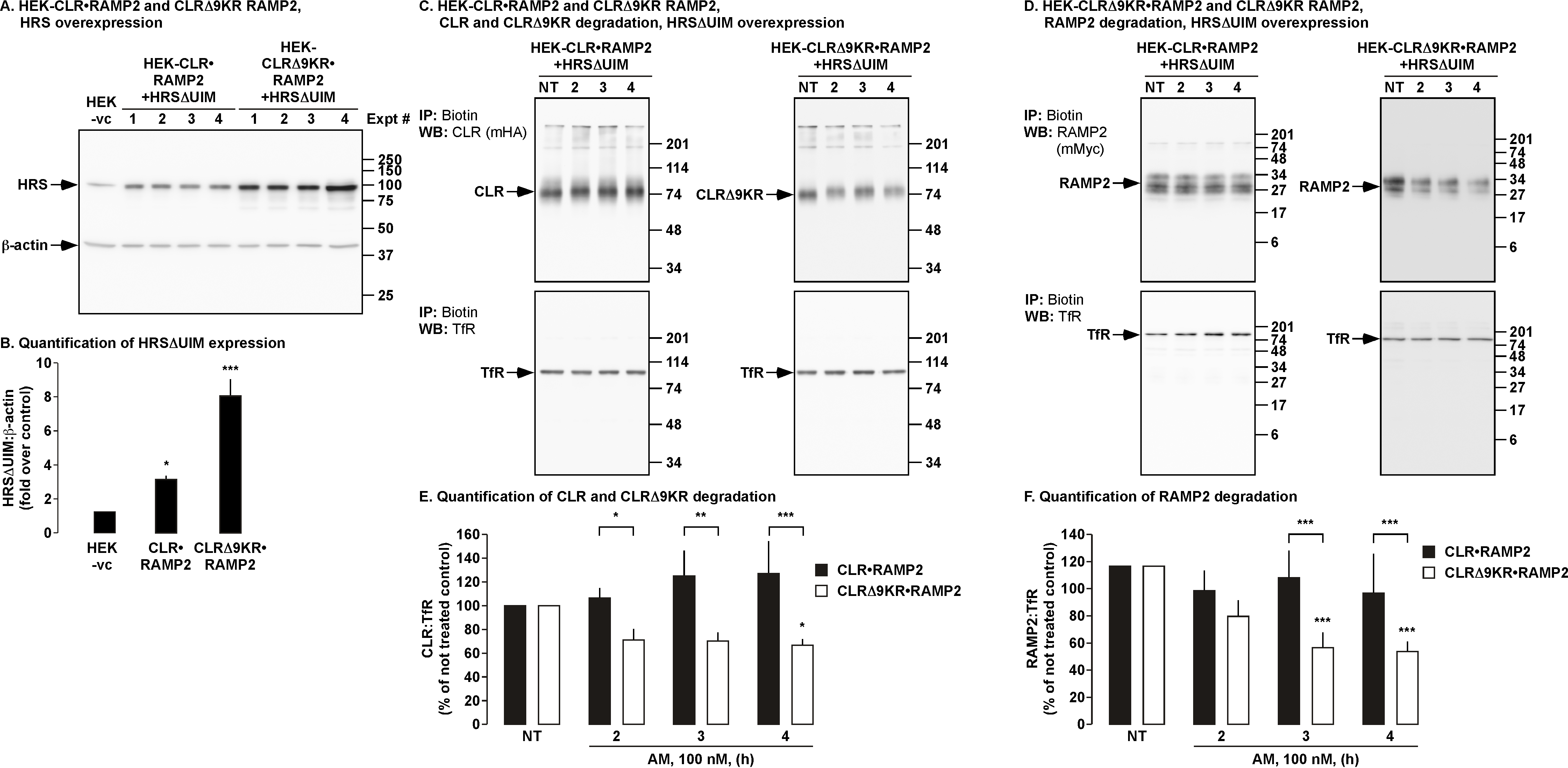
**

**Figure S10.** Effect of HRSΔUIM on the degradation of CLR, CLRΔ9KR and RAMP2. (**A, B**) Cell lysates from experimental cells were analysed by Western blotting and levels of HRSΔUIM and β-actin quantified. (**C, D**) Cell-surface biotinylated HEK-CLR•RAMP2 and HEK-CLRΔ9KR•RAMP2 cells expressing HRSΔUIM were not treated (NT) or challenged with AM (100 nM, 2-4 h), biotinylated proteins immunoprecipitated (IP) and Western blots (WB) probed for CLR (mouse-HA, mHA), CLRΔ9KR (mHA), RAMP2 (mouse-Myc, mMyc) and transferrin receptor (TfR, loading control). In untreated HEK-CLR•RAMP2 and HEK-CLRΔ9KR•RAMP2 cells, CLR, CLRΔ9KR, RAMP2 and TfR were readily detected. AM (100 nM, 4 h) induced degradation of CLR, CLRΔ9KR and RAMP2 to different levels. (**E, F**) Quantification of the degradation of CLR, CLRΔ9KR and RAMP2. n=4. Data were examined using ANOVA and Student-Newman-Keuls post-hoc test. **p*<0.05, ***p*<0.01, ****p*<0.001 compared to NT control or HEK-vc unless otherwise indicated.

**
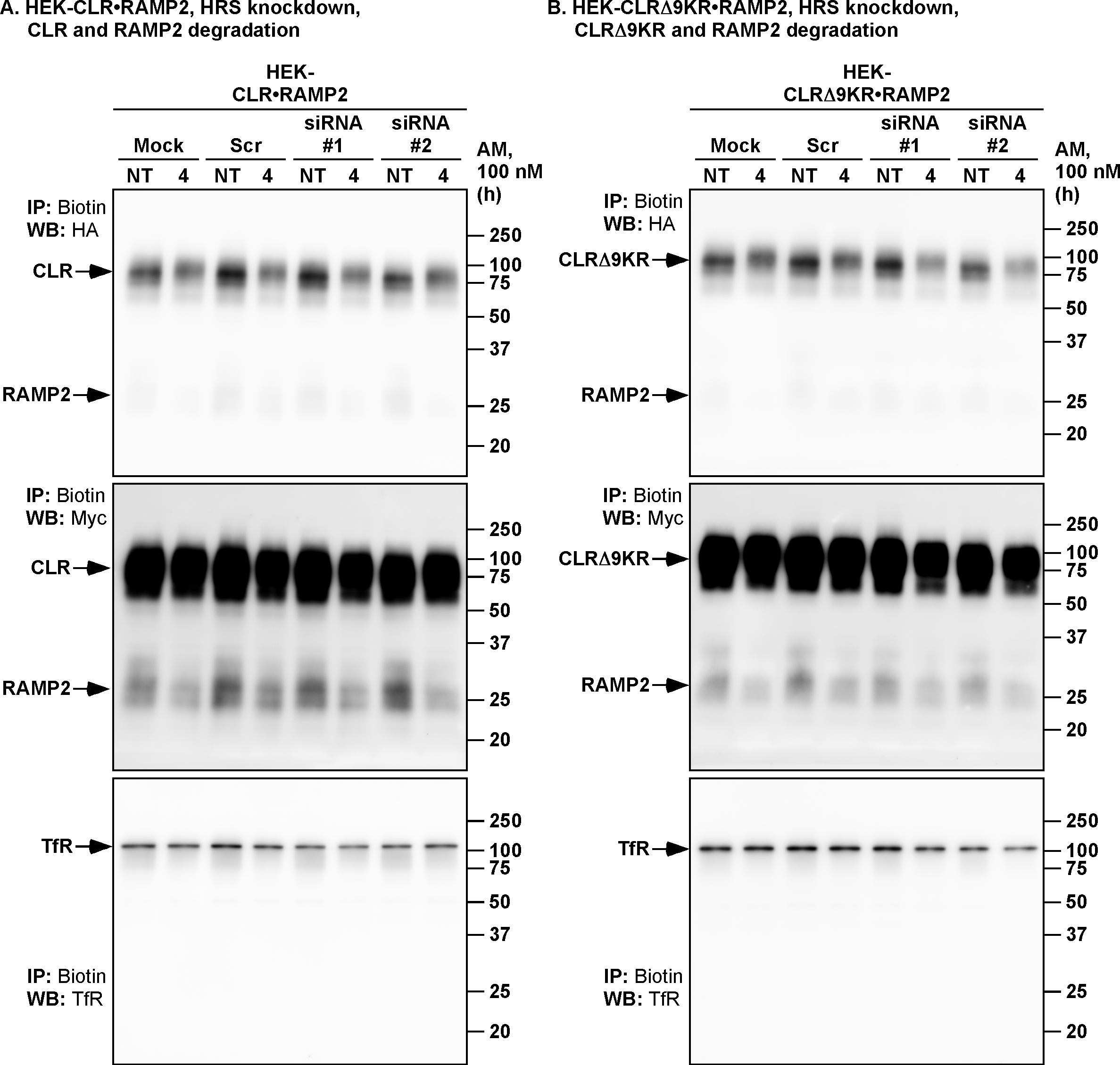
**

**Figure S11.** Effect of HRS knockdown on degradation of CLR, CLRΔ9KR and RAMP2. (**A, B)** Cell-surface biotinylated HEK cells expressing CLR•RAMP2 or CLRΔ9KR•RAMP2 and either mock transfected (Mock, control) or transfected with a non-targeting pool of siRNA (Scr) or siRNA to HRS (siRNA #1 and #2) were not treated (NT) or challenged with AM (100 nM, 4 h), biotinylated proteins immunoprecipitated (IP) and Western blots (WB) probed for CLR (mouse-HA, mHA), CLRΔ9KR (mHA), RAMP2 (mouse-Myc, mMyc) and transferrin receptor (TfR, loading control). In untreated HEK-CLR•RAMP2 and HEK-CLRΔ9KR•RAMP2 cells, CLR, CLRΔ9KR, RAMP2 and TfR were readily detected in all treatment types. AM (100 nM, 4 h) induced significant degradation of CLR, CLRΔ9KR and RAMP2 to similar levels in each cell type. n=4. This figure shows the full length blots in Fig. 8 (panels A and B) of the manuscript.

**
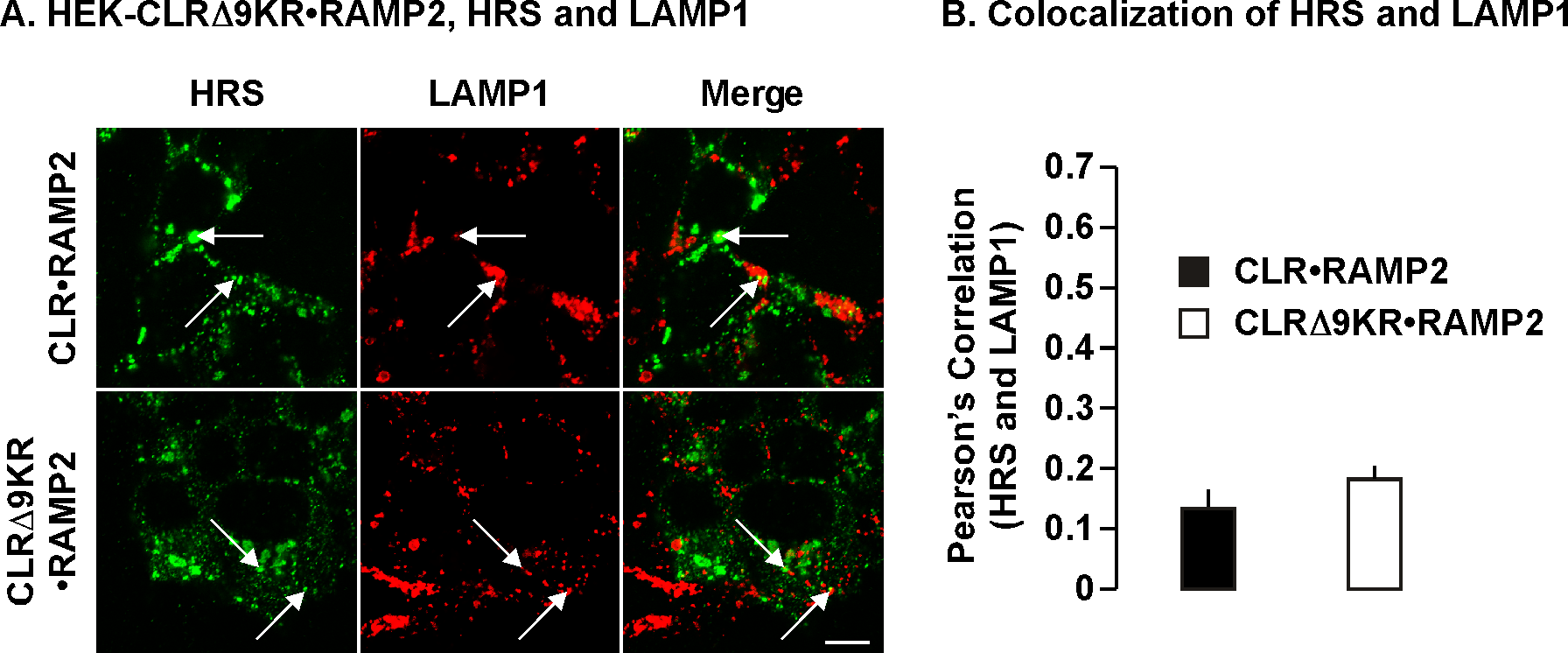
**

**Figure S12.** LAMP1 colocalizes with HRS in HRS overexpressing cells. (**A**) HEK-CLR•RAMP2 and HEK-CLRΔ9KR•RAMP2 cells were fixed, permeabilized and HRS and a marker for lysosomes (LAMP1) localized by immunofluorescence and confocal microscopy. In both CLR•RAMP2 and CLRΔ9KR•RAMP2 cells, HRS and LAMP1 were colocalized to similar extents (arrows). (**B**) Quantification of HRS and LAMP1 colocalization. n=3, Scale bar, 10 µm.

**Supplemental Methods**

**Reverse Transcription-PCR**

RNA from HMEC-1cells was isolated using Trizol (Invitrogen) and was reverse-transcribed using standard protocols with random hexamers and TaqMan reverse transcription reagents (Applied Biosystems, Carlsbad, CA, USA). Subsequent PCR reactions used primers specific for rat CLR and RAMP2 (CLR: forward, 5`-CGTGTTTGCAGAGAAGCAAC-3, reverse, 5`-GACCCTGGAAGTGCATAAGG-3`; RAMP2: forward, 5`-CTCAGCCTCTTCCCACCAC-3`, reverse, 5`-AGTTGGCAAAGTGGATCTGG-3`). Control reactions omitted reverse transcriptase. PCR products were separated by electrophoresis, stained with ethidium bromide, and sequenced to confirm identity.

**Flow Cytometry**

To examine cell-surface expression of CLR•RAMP2 and CLRΔ9KR•RAMP2, RAMP2 was labeled by incubating cells, with an antibody to the extracellular epitope tag (rabbit anti-c-Myc, 1:250; 30 min, 37°C) in DMEM-BSA. Cells were washed with PBS, stimulated with AM, detached from culture dishes by trypsinization, washed with PBS and incubated with donkey anti-rabbit DyLight 649 in DMEM-BSA (1 h, 4ºC). Cells were then washed and analyzed using a FACSCanto flow cytometer (BD Bioscience, Oxford, U.K.) and DIVA software. Only live cells were analyzed (propidium iodide exclusion).
